# Inter-embryo gene expression variability recapitulates the hourglass pattern of evo-devo

**DOI:** 10.1186/s12915-020-00842-z

**Published:** 2020-09-19

**Authors:** Jialin Liu, Michael Frochaux, Vincent Gardeux, Bart Deplancke, Marc Robinson-Rechavi

**Affiliations:** 1 Department of Ecology and Evolution, University of Lausanne, 1015 Lausanne, Switzerland; 2 Swiss Institute of Bioinformatics, 1015 Lausanne, Switzerland; 3 Laboratory of Systems Biology and Genetics, Institute of Bioengineering, School of Life Sciences, Ecole Polytechnique Fédérale de Lausanne (EPFL), Lausanne, Switzerland

**Keywords:** Expression variability, Hourglass, Evo-devo

## Abstract

**Background:**

The evolution of embryological development has long been characterized by deep conservation. In animal development, the phylotypic stage in mid-embryogenesis is more conserved than either early or late stages among species within the same phylum. Hypotheses to explain this hourglass pattern have focused on purifying the selection of gene regulation. Here, we propose an alternative—genes are regulated in different ways at different stages and have different intrinsic capacities to respond to perturbations on gene expression.

**Results:**

To eliminate the influence of natural selection, we quantified the expression variability of isogenetic single embryo transcriptomes throughout fly *Drosophila melanogaster* embryogenesis. We found that the expression variability is lower at the phylotypic stage, supporting that the underlying regulatory architecture in this stage is more robust to stochastic variation on gene expression. We present evidence that the phylotypic stage is also robust to genetic variations on gene expression. Moreover, chromatin regulation appears to play a key role in the variation and evolution of gene expression.

**Conclusions:**

We suggest that a phylum-level pattern of embryonic conservation can be explained by the intrinsic difference of gene regulatory mechanisms in different stages.

## Background

Both morphological and transcriptomic surveys have proposed an “hourglass” model of evo-devo [1, 2]. The mid-development phylotypic stage is more conserved than both early and late development [3–7]. Currently, the proposed mechanisms for this pattern are mainly based on how natural selection shapes the outcome of regulatory variation on gene expression. The first hypothesis interpreted high conservation as a result of negative selection [1, 2, 8]. For example, Raff suggested a high inter-dependence in signaling among developmental modules in middle development, so expression changes underlying this stage will generally be deleterious and under negative selection. An alternative hypothesis, however, argues that high conservation can also be the result of variation being less visible to positive selection [9, 10]. In this scenario, embryonic development is more likely to evolve when ecological niches demand it. For example, variation in early development can result from adaptation to diverse ecological circumstances [11]. To distinguish the two hypotheses, Zalts and Yanai [6] compared the expression variation of 20 *Caenorhabditis elegans* mutation accumulation strains across embryonic development and found that the nematode phylotypic stage has lower expression variation. Since mutation accumulation experiments mostly remove the effect of positive selection, this study indicates that positive selection is not necessary to obtain an hourglass pattern of expression evolution.

The hourglass pattern may also result from the regulatory mechanisms of genes at different stages having a different inherent tendency to respond to perturbations. Under this hypothesis, the outcome of regulatory mutations on gene expression would be biased, and this might impact the patterns of expression divergence between species, even in the absence of patterns of natural selection. For example, genes regulated by redundant enhancers are more robust to genetic variation on gene expression [12, 13]. In addition, broad promoters (which initiate transcription at dispersed regions) are more robust to mutations than narrow ones (which initiate transcription at precise positions) [14]. What is more, chromatin regulators can act as capacitors to buffer expression divergence between species [15]. Gene expression can vary among isogenic individuals in homogenous environments [16–20], suggesting widespread stochastic perturbations during transcription. For example, gene expression can be perturbed by the random distribution of molecules at cell division or by the inherent randomness of biochemical reactions with low molecule numbers [18, 21]. It has been suggested that mechanisms which confer robustness to environmental or stochastic perturbations on gene expression could also buffer the effects of genetic perturbations [22–24]. Under this hypothesis, we can use these stochastic perturbations of gene expression to estimate how expression responds to random genetic perturbations at different stages. If the phylotypic stage is more robust to genetic perturbations on gene expression, we should observe low inter-embryo expression variability at this stage, even among isogenic embryos in constant conditions.

In this study, we built a single embryo transcriptome time series across fly embryonic development, with a high number of isogenic replicates. Using this dataset, we investigated the developmental patterns of expression variability and found that the expression variability recapitulates an hourglass pattern, with the minimum of noise at extended germband, the phylotypic stage.

## Results

### Single embryo RNA-seq profile over embryogenesis

**Fig. 1.**
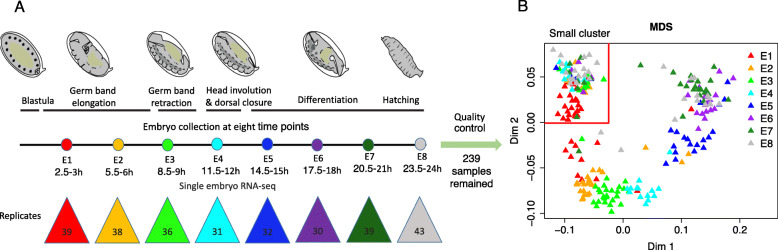
Studying the expression variability throughout embryogenesis. **a** Methods outline. We performed single embryo BRB-seq [25] at eight developmental stages, indicated by different colored dots. The number of samples collected at each stage is indicated in the colored triangles. Embryo images are adapted from Levin et al. [30]. **b** Multidimensional scaling analysis (MDS) of 239 high-quality samples. Different colors indicate different stages. The samples can be split into two groups: a small cluster in the top-left delimited by two red lines, and the remaining samples, which are organized according to the embryonic stage

We generated 288 single embryo 3′ end transcriptomes using bulk RNA barcoding and sequencing (BRB-seq) [25], at eight developmental stages covering the whole fly embryogenesis, with 3 h intervals (Fig. 1a). After quality control, 239 samples were kept (Additional file 1: Figure S1, S2). On average, we obtained over 5 million uniquely mapped reads of protein-coding genes per embryo. Based on multidimensional scaling analysis (MDS), 150 embryos follow the developmental trajectory, while there is a small cluster of 89 embryos collected at different time points mixed together (Fig. 1b). The samples in this cluster appear to be unfertilized eggs (Additional file 1: Figure S3). All further analysis was performed only on the 150 fertilized embryos.

### The phylotypic stage is robust to stochastic perturbations on gene expression

**Fig. 2.**
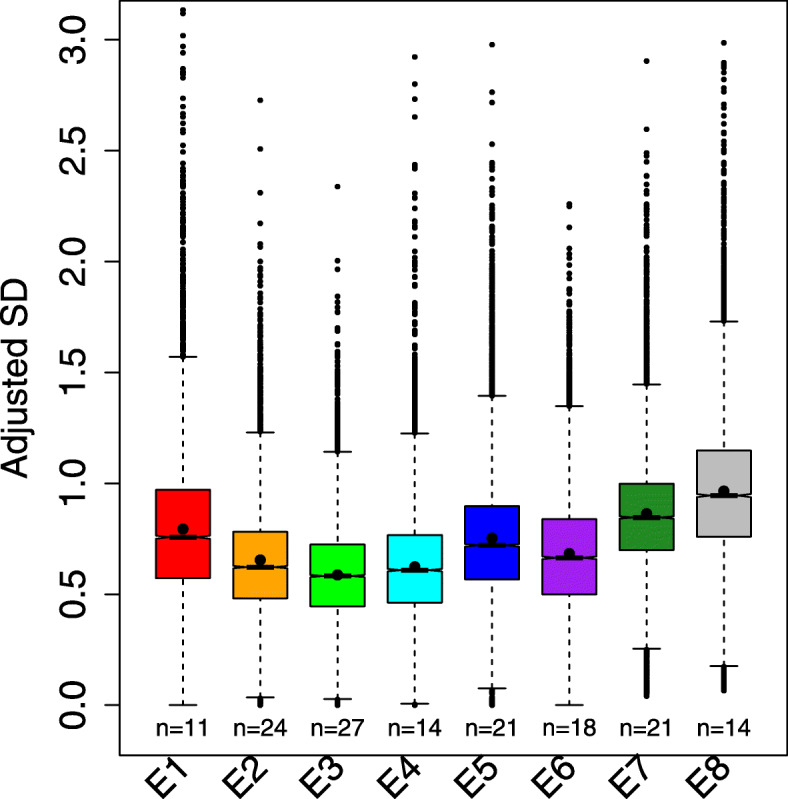
The phylotypic stage (E3) has lower expression variability. The number of individual samples in each development stage is indicated below each box. The lower and upper intervals indicated by the dashed lines (“whiskers”) represent 1.5 times the interquartile range (IQR), and the box shows the lower and upper intervals of IQR together with the median. The black dot in each box indicates the mean. We performed pairwise Wilcoxon tests between any two stages to test the significance. The multiple test corrected *p* values (Benjamini-Hochberg method) are shown in Additional file 2: Table S1; they are all < 10^−7^

We measured expression variability as adjusted SD and standard deviation (SD) of expression between replicates corrected for expression level (the “Materials and methods” section; Additional file 1: Figure S4-6). This expression variability follows an hourglass pattern overall, with a global minimum at E3 (Fig. 2), corresponding to the phylotypic stage of flies [7]. There is also a local minimum at E6. This is consistent with the pattern of transcriptome divergence between fly and mosquito *Anopheles gambiae*, with the global minimum at E3 and a local minimum at E6 [26]. We did not find any significant functional enrichment for genes which follow the hourglass variability pattern. Essential genes, and highly connected genes, have lower variability (Additional file 1: Figure S7), as observed in yeasts [27, 28], which indicates that variability is generally detrimental. Overall, expression variability is not equally distributed throughout embryogenesis, and the variability is lower at the phylotypic stage, supporting the hypothesis that the regulatory mechanisms at the phylotypic stage are more robust to stochastic perturbations on gene expression.

### Testing potential confounding factors of hourglass variability pattern

Our observations are robust to the use of different variability metrics (Additional file 1: Figure S8). Based on bootstrap analysis (“Materials and methods” section), we confirmed that all the stages, excepting E4, are robust to the choice of samples used to calculate noise (Additional file 1: Figure S9). Bootstrap results also suggest that the minimum variability extends over E3 to E4. To check whether high variability at E1 is related with a low number of embryos at this stage, we sampled the same number of embryos for each stage (8 embryos, without replacement) and re-calculated the median variability across genes. We repeated this process 500 times and compared the median of variability over development. The pattern is similar, with a minimum at E3 (Additional file 1: Figure S10). Another potential source of bias is maternal transcripts. The embryo transcriptome is dominated by zygotic transcripts as soon as 2.5 h after egg laying [29], so the high variability in E1 and E2 is not directly caused by maternal transcripts; we confirmed this by removing all maternally expressed genes (Additional file 1: Figure S11). Finally, the variation in the expression variability could either be due to changes in the set of active genes, with genes differing in their intrinsic variability levels, or to genome-wide changes in the regulation of variability. To test this, we reproduced our results restricted to the subset of genes which are expressed at all stages. Under the first explanation, we would expect to lose the hourglass variability pattern, but the pattern is maintained (Additional file 1: Figure S12), suggesting that the lower variability at E3 is due to genome-wide regulation mechanisms more than to changes in the gene set.

### Mutational robustness shapes the hourglass expression divergence pattern

**Fig. 3.**
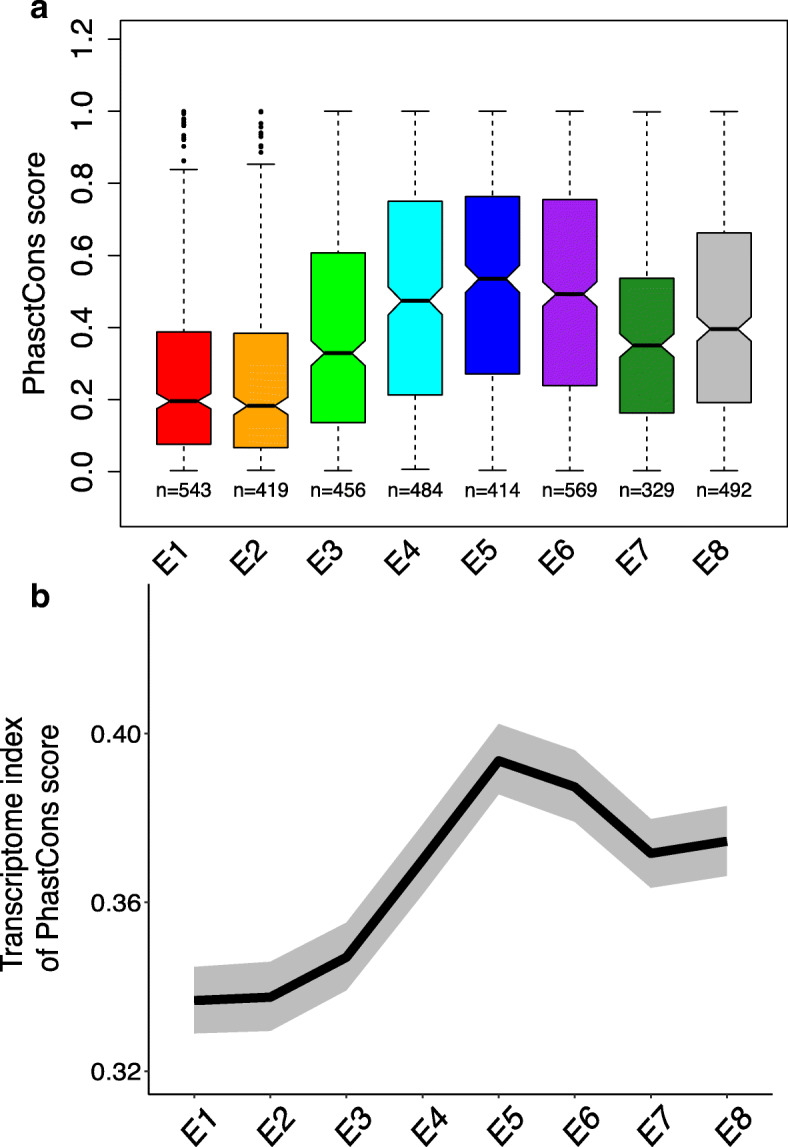
Promoter sequence conservation across embryogenesis. **a** Variation of promoter sequence conservation for stage-specific genes. Higher phastCons score means higher conservation. The lower and upper intervals indicated by the dashed lines (“whiskers”) represent 1.5 times the interquartile range (IQR), and the box shows the lower and upper intervals of IQR together with the median. The number of genes in each development stage is indicated below each box. The multiple test corrected *p* values (Benjamini-Hochberg method) between any two stages are shown in Additional file 2: Table S2. **b** Transcriptome index of promoter phastCons score across development. The gray area indicates 95% confidence interval

Several studies have suggested that mechanisms which confer robustness to stochastic variations can also buffer the effects of genetic variations [22–24]. This raises the possibility that the regulatory mechanisms at the phylotypic stage might also be characterized by stronger robustness to genetic perturbations on gene expression. In this scenario, we should expect relatively low regulatory sequence conservation for the stage with high gene expression conservation [7]. Indeed, mutations that are buffered would behave nearly neutrally. To test this hypothesis, we identified genes specifically expressed in each stage and compared sequence conservation of their core promoter regions between species (49 bp upstream transcription start site (TSS) to 10 bp downstream of the TSS as defined by Dreos et al. [31]; phastCons score [32]). We found that genes specific of E3 have a relatively weak promoter sequence conservation (Fig. 3a). This pattern remains using 200-bp or 400-bp regions around TSS but disappears using 1-kb regions (Additional file 1: Figure S13). Using transcriptome indexes of conservation (mean promoter sequence conservation weighted by expression), we can extend this observation to the full transcriptome (Fig. 3b). E3 has a relatively low index, again showing that genes expressed at this stage tend to have low promoter sequence conservation. These results support a role of buffering effects on regulatory mutations in the hourglass pattern of expression divergence in fly embryogenesis.

### Histone modifications and expression variability

**Fig. 4.**
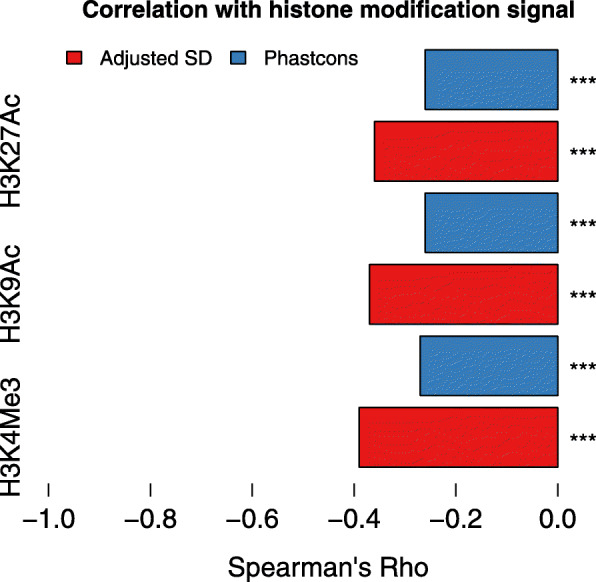
Histone modification signal, expression variability, and promoter sequence conservation. Red represents Spearman’s correlation coefficient between histone modification signal and expression variability; blue represents spearman’s correlation coefficient between histone modification signal and promoter sequence conservation. Here, for each gene, both its variability and its histone modification signal are the mean value across stages. ***p value < 10^−16^

To investigate the mechanisms which minimize expression variability, we focused on histone modifications. It has been suggested that histone modifications play a prominent role in regulating expression variability between cells (cell-to-cell expression variability) [33, 34, 35–37], between individuals (individual-to-individual expression variability) [19], and between environments (expression plasticity) [38]. To systematically study the role histone modifications play in relation to expression variability between embryos, we analyzed three active histone modifications (H3K4Me3, H3K9Ac, and H3K27Ac) at six developmental stages from modENCODE [39]. For each gene, we calculated the mean modification signal (background-subtracted tag density) of its proximal promoter region (2 kb upstream to 2 kb downstream for TSS). We found that higher modification signal genes tend to have lower variability for all histone modifications (Fig. 4), supporting a role in minimizing expression variability.

It has been found that, relative to narrow promoters, broad promoters were highly enriched with H3K9Ac [40], and Additional file 1: Figure S14). In addition, genes with broader promoters tend to have lower expression variability [41], and Additional file 1: Figure S15). These observations indicate that the negative correlation between histone modification signal and expression variability could be explained by promoter shape. After controlling the effects of promoter shape, however, we still found a strong negative correlation (Spearman’s rho − 0.34, − 0.32, − 0.32 for H3K4Me3, H3K9Ac, and H3K27Ac, respectively). Interestingly, the correlation between promoter shape index and expression variability is much weaker (Spearman’s rho 0.08), after controlling for histone modification signal.

### Histone modifications and promoter sequence conservation

To study the relationship between histone modifications and promoter sequence evolution, we analyzed the correlation between histone modification signal and promoter sequence conservation. We found that genes with higher histone modification signal tend to have less conserved core promoter regions between species (Fig. 4). Since histone modifications are enriched in highly expressed genes [42], and selective pressure against mutations changing gene expression level is stronger in highly expressed genes [43], genes with higher histone modification signal might be expected to have more conserved promoter sequence conservation. Our observation of the inverse pattern suggests that genes with higher histone modification signal are under stronger buffering effect, thus less sensitive to mutations in their regulatory regions, and are thus less conserved.

### Higher histone modification signal at the phylotypic stage

**Fig. 5.**
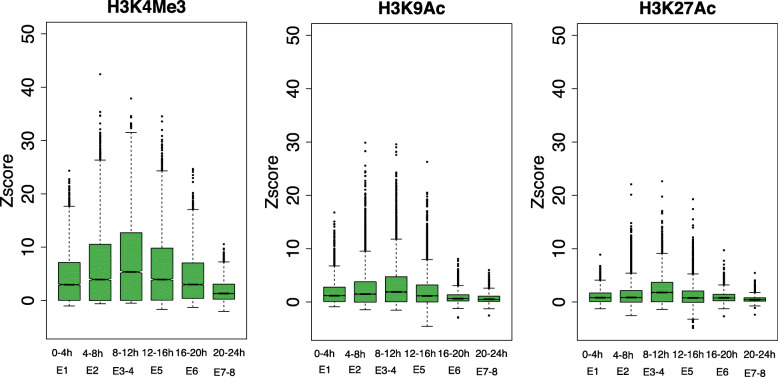
Histone modification signal (*Z* score relative to intergenic signal) across embryogenesis. Corresponding stages of our expression variability data are indicated below. The lower and upper intervals indicated by the dashed lines (“whiskers”) represent 1.5 times the interquartile range (IQR), and the box shows the lower and upper intervals of IQR together with the median. The multiple test corrected *p* values (Benjamini-Hochberg method) between any two stages are shown in Additional file 2: Table S3-S5

The relation between histone modifications and expression variability raises the possibility that the pattern of expression variability across development could be partially driven by changes in histone modification signal. To compare histone modification signal between stages, we normalized the promoter signal by that on intergenic regions (the “Materials and methods” section), which are not expected to change histone modification signal between stages. All normalized histone marks present an hourglass-like pattern, with the highest signal at 8–12 h, corresponding to E3 and E4, i.e., the lowest expression variability (Fig. 5). Generally, these results show that histone modification signal changes over development, with a correspondence between stronger histone modification signal and lower expression variability, consistent with our hypothesis.

## Discussion

In this work, we found an uneven distribution of isogenic inter-individual expression variability, and thus of robustness of the process of gene expression, across development. This mirrors the hourglass evo-devo pattern [1, 2]. Stage E3 is the most robust to a stochastic variation on gene expression, with lower expression variability, and is the phylotypic stage of fly, with a conservation between species [7].

The hourglass pattern has been mainly interpreted as a signal of increased negative selection on gene regulation at mid-development, the phylotypic stage [1, 2, 4, 6, 8], although it may also result from an increased positive selection at both early and late development [6, 9, 10, 44]. However, our finding that genes expressed at distinct stages show distinct patterns of variability suggests an alternative, although not exclusive, model: genes expressed at the phylotypic stage utilize different regulatory strategies, which respond differently to perturbations. Thus, we should not expect divergence in gene expression to be equally likely at all stages even without natural selection on this divergence. The natural selection, instead, could be in part in the control of expression variability in individuals.

Consistent with previous observations in *Arabidopsis thaliana* [19], we found that histone modification is an important determinant of expression variability between multicellular individuals. Interestingly, histone modification also plays a role in regulating expression variability between single cells [33, 34, 35–37]. For example, a gene’s expression variability can be changed by modulating histone acetylation level of its promoter [35]. In addition, histone modifications are also associated with expression variability between environments [38]. In line with these results, we found that higher histone modification genes have less conserved promoter sequences, suggesting that histone modification might also buffer genetic variations on gene expression. Although there is no direct experimental validation, it has been found that deleting chromatin regulators tends to increase the expression divergence between species [15]. Since the sources of expression variability discussed above are quite different, the chromatin structure appears to be an important regulator of the robustness of gene expression under different types of perturbations.

While we found that genes specific of embryonic stages with lower expression divergence (E3, E4, E5, E6) generally have higher sequence conservation of promoters, the phylotypic stage (E3) does not have the highest promoter sequence conservation, suggesting that the lowest expression divergence at this stage is driven at least in part by mutational robustness. Our analyses have two potential caveats. First, our results were based solely on promoter analysis, and it remains to be seen how much these observations extend to other regulatory elements. Second, we only considered nucleotide substitution changes, but not promoter turnover. For large effect mutations, such as insertion, deletion, and turnover, purifying selection may be more efficient than mutational robustness to keep conserved expression level. For example, Piasecka et al. [45] found the neighborhood of genes specifically expressed in the phylotypic stage of zebrafish to be enriched with transposon free regions and long conserved non-coding elements, indicating stronger purifying selection for large effect mutations in the phylotypic stage. So, it is possible that both purifying selection and mutational robustness together shape the lower expression divergence in the fly phylotypic stage.

Although mutational robustness can evolve under natural selection theoretically [46], the conditions are too restrictive to be relevant in practice. In contrast, selection for robustness to environmental or stochastic variations can have a clear fitness advantage (individual-based and immediate). Thus, we propose that mutational buffering is a by-product of selection for minimizing such expression variability. The exact roles of natural selection and of histone modifications in the patterns that we observe remain to be tested, as does the generality of our observations beyond *D. melanogaster*. Our results support an important role for the control of expression variability in embryonic gene expression and in the evolution of development. We propose that selection for robustness to stochastic and to environmental perturbations in a key embryonic stage has led to the evolutionary conservation over large time scales which characterizes the phylotypic stage.

## Conclusions

Overall, we suggest that the phylotypic stage is characterized by selection for robustness to stochastic and environmental perturbations. This could lead to mutational robustness, thus evolutionary conservation of expression and the hourglass pattern.

## Materials and methods

### Embryo collection and RNA extraction

Fly lines (w^1118^) were obtained from the Bloomington Stock Center and reared at room temperature on a standard fly medium with a 12-h light-dark cycle. The fly medium we used is composed of 6.2 g agar powder (ACROS N. 400400050), 58.8 g Farigel wheat (Westhove N. FMZH1), 58.8 g yeast (Springaline BA10), 100 mL grape juice; 4.9 mL propionic acid (Sigma N. P1386), 26.5 mL of methyl 4-hydroxybenzoate (VWR N. ALFAA14289.0) solution (400 g/L) in 95% ethanol, and 1 L water. One hundred to 150 flies were transferred to cages, which were sealed to a grape agar plate (1:1 mixture of 6% agar and grape juice). We used 4 separate cages to collect the embryos. The adults were kept overnight on this plate before being transferred to a new plate supplemented with yeast paste. Synchronization of eggs on this plate lasted for 2 h before being swapped with a new plate supplemented with yeast paste. We let the adults lay eggs for 30 min before removing the plate and letting the eggs incubate for the desired time.

Eggs were harvested using the following protocol. First a 1:1 bleach (Reactolab 99412) 1× PBS mix was poured on the plate and incubated for 2 min. During this incubation, we used a brush to lightly scrape the surface to mobilize the embryos. We then poured the PBS-bleach mixture through a sieve, washed the plate with 1× PBS, and poured the wash on the same sieve. We washed the sieve several times with 1× PBS until the smell of bleach disappeared. Single embryos were then manually transferred to Eppendorf containing 50 μL beads and 350 μL TRIzol (Life Technologies AM9738). The tubes were homogenized in a Precellys 24 Tissue Homogenizer at 6000 rpm for 30 s. Samples were then transferred to liquid nitrogen for flash freezing and stored at − 80 °C. For RNA extraction, tubes were thawed on ice, supplemented with 350 μL of 100% ethanol before homogenizing again with the same parameters. We then used the Direct-zol™ RNA Miniprep R2056 Kit, with the following modifications: we did not perform DNAse I treatment, we added another 2 min centrifugation into an empty column after the RNA Wash step, finally elution was performed by adding 8 μL of RNAse-free water to the column, incubation at room temperature for 2 min, and then centrifugation for 2 min. RNA was transferred to a low-binding 96-well plate and stored at − 80 °C.

### Bulk RNA barcoding and sequencing

The BRB-seq is a technique for multiplexed RNA-seq [25] which is able to provide high-quality 3′ transcriptomic data at a low cost (e.g., 10-fold lower than Illumina Truseq Stranded mRNA-seq). The data (fastq files) generated from BRB-seq are multiplexed and asymmetrical paired reads. Read R1 contains a 6-bp sample barcode, while read R2 contains the fragment sequence to align to the reference genome.

#### Library preparation

RNA quantity was assessed using picogreen (Invitrogen P11496). Samples were then grouped according to their concentration in 96-well plates and diluted to a final concentration determined by the lowest sample concentration on the plate. RNA was then used for gene expression profiling using BRB-seq. In short, the BRB-seq protocol starts with oligo-dT barcoding, without TSO for the first-strand synthesis (reverse transcription), performed on each sample separately. Then, all samples are pooled together, after which the second strand is synthesized using DNA PolII Nick translation. The sequencing library is then prepared using cDNA augmented by an in-house-produced Tn5 transposase preloaded with the same adapters (Tn5-B/B), and further enriched by limited-cycle PCR with Illumina compatible adapters. Libraries are then size-selected (200–1000 bp), profiled using High Sensitivity NGS Fragment Analysis Kit (Advanced Analytical, #DNF-474), and measured using Qubit dsDNA HS Assay Kit (Invitrogen, #Q32851). In total, we generated four libraries. For details of the library information, please check Additional file 2: Table S13.

#### Sequencing

Libraries were mixed in equimolar quantities and were then sequenced on an Illumina Hi-Seq 2500 with pair-end protocol (read R2 with 101 bp) at the Lausanne Genomic Technologies Facility.

### RNA-seq analysis

#### Generating expression matrix

The fastq files were first demultiplexed by using the “Demultiplex” tool from BRB-seqTools suite (available at https://github.com/DeplanckeLab/BRB-seqTools). Then, we trimmed the polyA sequences of the demultiplexed files by using the “Trim” tool. Next, the STAR aligner [47] was used to map the trimmed reads to the reference genome of fly *Drosophila melanogaster* (BDGP6, Ensembl release 91 [48]). Finally, the read count of each gene was obtained with HTSeq [49].

#### Filtering samples and genes

Low-quality samples need to be filtered out, since they might bias the results of downstream analyses. In order to assess sample quality, we calculated the number of uniquely mapped reads and of expressed genes for each sample [50]. We removed samples with < 0.3 million uniquely mapped reads or with < 4500 expressed genes (Additional file 1: Figure S1). We confirmed that these filtered samples are indeed outliers in a multidimensional scaling analysis (MDS) (Additional file 1: Figure S16). Since lowly expressed genes have a larger technical error, to minimize the technical noise, we need to remove lowly expressed genes as well. We first calculated counts per million (cpm) with the edgeR package [51] for each gene. Then, we removed genes with mean cpm across samples ≤ 1, as suggested by Lun et al. [50]. Finally, for the remaining genes, we re-transformed their cpm values to the original count values for the downstream normalization analysis. After filtering, we obtained an expression count matrix with 239 samples (Additional file 1: Figure S2) and 8004 protein-coding genes.

#### Normalization and batch effect correction

Because BRB-seq retains only the 3′ end of the transcript, we performed sample normalization by using quantile normalization with log transformation in the voom package [52], but without transcript length normalization. To remove potential batch effects across the four libraries, we applied the ComBat function in the sva package [53] to the normalized and log2 transformed expression data. For genes with expression values less than 0 after Combat, or with original expression values equal to 0, we change its values to 0 after Combat correction as suggested by Kolodziejczyk et al. [54].

### Multidimensional scaling analysis

A number of factors could be invoked to explain the two groups observed in our multidimensional scaling analysis (MDS) (Fig. 1b), but they should also explain that only one group is structured according to the developmental time. The obvious hypothesis that they correspond to male and female embryos does not explain that structure and is also not supported by examining X/autosome gene expression ratios (Additional file 1: Figure S17). An alternative hypothesis is that samples in the small cluster are unfertilized eggs. If an egg is not fertilized, after completion of meiosis, the development will be arrested [55], but they are visually indistinguishable. This hypothesis is confirmed by at least two lines of evidence, in addition to the lack of developmental time structure. First, for expression correlation, all samples in the small cluster are highly correlated with unfertilized egg, while the correlations in the other samples gradually decrease with development (Additional file 1: Figure S3A). Second, all the samples from the small cluster are enriched with meiosis-related genes (Additional file 1: Figure S3B). Thus, we excluded the small cluster for downstream analyses, i.e., we used 150 embryos with an average of 18 individuals per developmental stage.

### Metrics of expression variability

Expression variability is generally measured by the coefficient of variation (CV) [56]. However, a gene’s CV is strongly dependent on its RNA abundance (Additional file 1: Figure S4). While this is an inherent property of time-interval counting processes (such as a Poisson process), it makes the comparison of variability between different conditions difficult [54, 57]. Distance to median (DM, the distance between the squared CV of a gene and its running median) has been proposed as a variability metric that is independent of expression level [28, 54, 57]. However, the DM is still strongly negatively correlated with the mean expression level in our data (Additional file 1: Figure S5). To avoid this dependency, we developed another variability measure, the adjusted standard deviation (adjusted SD), by calculating the ratio between observed SD and expected SD. Following the same approach as Barroso et al. [58], we performed polynomial regressions to predict the expected SD from mean expression. Since the adjusted SD metric works much better than DM in terms of accounting for the confounding effects of mean expression (Additional file 1: Figure S6), we adopted it as a measure of expression variability in our study. As observed in yeasts [27, 28], we found that essential genes and hubs (proteins in the center of protein-protein interaction network) have lower expression variability than other genes (Additional file 1: Figure S7), indicating selection to reduce it. This observation provides a control that we are indeed measuring biologically relevant expression variability.

Detailed calculation of expression variability is as follows:

#### Adjusted SD

For each gene, we computed the standard deviation (SD) in each stage and over all stages. Then, we fitted a polynomial model to predict the global (across all stages) SD from the global mean expression. We increased the degrees of the model until there was no more significant improvement (tested with ANOVA, *p* < 0.05 as a significant improvement). Then, based on this best-fitting model, for each gene, we computed its predicted global SD based on its global mean expression. Finally, the adjusted SD of a gene in one stage is this gene’s SD in its corresponding stage divided by its predicted global SD. This method is derived from Barroso et al. [58], but computing adjusted SD rather than adjusted variance.

#### Distance to median: the distance between the squared coefficient of variation of a gene and its running median

For each gene, we computed its squared CV in each stage and over all stages. Then, we ordered genes based on their global (across all stages) mean expression. Next, we defined a series of sliding windows of 50 genes with 25 genes overlap, starting from the lowest global mean expression. Finally, the distance to median of a gene in one stage is the stage-specific log10 squared CV minus the median of global log10 squared CV in this gene’s corresponding window. R code was modified from the DM function of the scran package [50].

### Bootstrap analysis

For each stage, we randomly sampled the same number of samples. Then, we computed the adjusted SD based on these random samples. We repeated the first two steps 500 times. Each time, we only kept the median of the adjusted SD for each stage. Thus, in each stage, we obtained 500 medians. Finally, we performed a Wilcoxon test to test the significance of the difference between the bootstrapped values of different stages.

### ChIP-seq data analysis

#### Histone modification signal datasets

The signal data files of four euchromatin histone modification marks (H3K4me3, H3K9ac, and H3K27ac) at six developmental stages (0–4 h, 4–8 h, 8–12 h, 12–16 h, 16–20 h, 20–24 h) were downloaded from modENCODE [39] (NCBI GEO: GSE16013) (March 2018). The signal is smoothed, background-subtracted tag density. The signal was precomputed along the genome in 35-bp windows.

#### Histone modification signal for promoter

For each gene, we calculated the mean signal of its proximal promoter (2 kb upstream to 2 kb downstream for transcription start site (TSS)) by using the bedtools “map” command [59]. The TSS and transcription end site (TES) information was retrieved from Ensembl release 91 [48]. For a gene with several TSS and TES, we use its mean coordinates.

#### Histone modification signal *Z* score transformation

For each modification mark in each stage, the signal value was transformed into a *Z* score by subtracting the mean signal across intergenic regions and dividing by the standard deviation signal of the intergenic regions. The intergenic region was defined by removing all proximal promoter regions and gene body regions (TSS to TES) with the bedtools “subtract” command [59]. Since the three histone modification marks are largely enriched at promoter over intergenic regions in Drosophila [39], this allows to normalize between libraries. Then, for each gene, we re-calculated the mean signal (*Z* score) of its proximal promoter (2 kb upstream to 2 kb downstream for TSS) by using the bedtools “map” command [59].

### Identification of stage specifically expressed genes

Following the same approach as previously [10], we first defined 8 stage-specific expressed artificial expression profile (Additional file 1: Figure S18A). Then, for each gene, we performed Pearson’s correlation between its real expression and this artificial expression. Finally, for each artificial expression profile, we kept genes with top 10% correlation coefficient as the corresponding stage specifically expressed genes (Additional file 1: Figure S18B).

### Identification of hourglass expression variability genes

Similar to the stage specifically expressed gene identification approach, we correlated each gene’s variability profile with the median across all genes. Then, we kept genes with the top 10% correlation coefficient as the ones following the global hourglass variability profile.

### Gene Ontology enrichment analysis

We performed Gene Ontology (GO) enrichment analysis for hourglass expression variability genes by using the topGO [60] R package with the “elim” algorithm.

### Partial correlation

The R package “ppcor” [61] was used to compute Spearman’s partial correlation coefficient between histone modification signal and expression variability after controlling for the effect of promoter shape.

### Transcriptome index analysis

A “transcriptome index” [62, 63] is a weighted mean of a feature over all genes, where the weights are the expression levels of the genes at each condition (e.g., developmental stage). The transcriptome index of phastCons was calculated as:

$$ {\mathrm{TPI}}_s=\frac{\sum \limits_{i=l}^n{\mathrm{phastCons}}_i\times {e}_{is}}{\sum \limits_i^n{e}_{is}} $$

where *s* is the developmental stage, phastCons_*i*_ is the promoter sequence conservation score of gene *i*, *n* is the total number of genes, and *e*_*is*_ is the expression level (log-transformed) of gene *i* in developmental stage *s*.

### Confidence interval analysis for transcriptome index

Firstly, we randomly sampled gene IDs from each original data set 10,000 times with replacement. Then, we computed transcriptome indexes for the 10,000 samples. Finally, the 95% confidence interval is defined as the range from quantile 2.5% to quantile 97.5% of the 10,000 transcriptome indexes.

### Meiosis-related genes and transcription factors

The meiosis-related genes and transcription factors were downloaded from AmiGO [64] (May 2018).

### Individual unfertilized eggs RNA-seq data

The normalized and log-transformed expression matrix of individual unfertilized eggs was downloaded from NCBI GEO: GSE68062 [65] (May 2018).

### Promoter shape index

The promoter shape index was downloaded from [66]. (June 2019). Lower value means broader promoter.

### Essential gene annotation and protein connectivity datasets

The gene essentiality and protein connectivity datasets were downloaded from OGEE v2 [67] (March 2018).

### PhastCons score

The pre-computed sequence conservation score phastCons [32] of fly genome was downloaded from http://hgdownload.soe.ucsc.edu/goldenPath/dm3/phastCons15way/ (February 2018). Higher value means higher conservation.

### Experimentally validated core promoter regions

Experimentally validated transcription start sites (TSSs) were downloaded from the Eukaryotic Promoter Database (EPD) [31] (May 2018). For a gene with several TSSs, we selected the most representative one (the TSS that has been validated by the largest number of samples). The core promoter region was defined as 49 bp upstream TSS to 10 bp downstream of the TSS [31]. We used EPD-defined TSSs here because they are more accurate for defining core promoters, whose function is expected to be related to sequence conservation. Whereas for histone modification signal for promoter, we used Ensembl-defined TSSs to be consistent with the source of transcription end site (TES) information, and precision was less important in defining broader proximal promoters.

## Supplementary information

**Additional file 1:** **Figure S1-S18.** **Figure S1.** Relationship between uniquely mapped reads and expressed genes. **Figure S2.** Proportion of retained samples in each development stage. **Figure S3.** Evidence that the samples from the small cluster are unfertilized eggs. **Figure S4.** Relationship between average expression and coefficient of variation at each stage. **Figure S5.** Relationship between average expression and distance to median at each stage. **Figure S6.** Relationship between average expression and adjusted SD at each stage. **Figure S7**. Relationship between expression variability and protein importance. **Figure S8.** Variation of expression variability across development using alternate measures of variability. **Figure S9.** Bootstrap analysis of the variability calculation. **Figure S10.** Random sampling analysis of expression variability. **Figure S11.** Expression variability pattern for genes without maternally expressed genes. **Figure S12.** Expression variability pattern for genes expressed at all stages. **Figure S13**. Promoter sequence conservation across embryogenesis for different definitions of promoter width. **Figure S14**. Relationship between promoter shape index and histone modification signal. **Figure S15.** Relationship between promoter shape index and expression variability. **Figure S16.** Multidimensional scaling analysis for all samples. **Figure S17.** Mapping of X/autosome gene expression ratios to the multidimensional scaling analysis plot. **Figure S18.** Detection of stage specific genes.

**Additional file 2:** **Tables. S1-S13.** **Table S1-S12.** multiple test corrected *p*-values of variability (adjusted SD) comparison between any two stages. **Table S13.** RNA-seq library information.

## Data Availability

Data files and analysis scripts are available on GitHub: https://github.com/ljljolinq1010/expression-noise-across-fly-embryogenesis. Expression datasets have been deposited to the Gene Expression Omnibus with accession number GSE128370 [68].

## References

[1] Raff RA. The shape of life: genes, development, and the evolution of animal form. Chicago: University of Chicago Press. 1996.

[2] Duboule D (1994). Temporal colinearity and the phylotypic progression: a basis for the stability of a vertebrate Bauplan and the evolution of morphologies through heterochrony. Development..

[3] Irie N, Kuratani S (2011). Comparative transcriptome analysis reveals vertebrate phylotypic period during organogenesis. Nat Commun.

[4] Hu H, Uesaka M, Guo S, Shimai K, Lu T-M, Li F (2017). Constrained vertebrate evolution by pleiotropic genes. Nat Ecol Evol..

[5] Levin M, Hashimshony T, Wagner F, Yanai I (2012). Developmental milestones punctuate gene expression in the Caenorhabditis embryo. Dev Cell.

[6] Zalts H, Yanai I (2017). Developmental constraints shape the evolution of the nematode mid-developmental transition. Nat Ecol Evol.

[7] Kalinka AT, Varga KM, Gerrard DT, Preibisch S, Corcoran DL, Jarrells J (2010). Gene expression divergence recapitulates the developmental hourglass model. Nature..

[8] Galis F, Metz JA (2001). Testing the vulnerability of the phylotypic stage: on modularity and evolutionary conservation. J Exp Zool.

[9] Darwin C. The descent of man, and selection in relation to sex. Princeton: Princeton University Press. 1871.

[10] Liu J, Robinson-Rechavi M (2018). Adaptive evolution of animal proteins over development: support for the Darwin selection opportunity hypothesis of evo-devo. Mol Biol Evol.

[11] Kalinka AT, Tomancak P (2012). The evolution of early animal embryos: conservation or divergence?. Trends Ecol Evol.

[12] Frankel N, Davis GK, Vargas D, Wang S, Payre F, Stern DL (2010). Phenotypic robustness conferred by apparently redundant transcriptional enhancers. Nature..

[13] Perry MW, Boettiger AN, Bothma JP, Levine M (2010). Shadow enhancers foster robustness of Drosophila gastrulation. Curr Biol.

[14] Schor IE, Degner JF, Harnett D, Cannavò E, Casale FP, Shim H (2017). Promoter shape varies across populations and affects promoter evolution and expression noise. Nat Genet.

[15] Tirosh I, Reikhav S, Sigal N, Assia Y, Barkai N (2010). Chromatin regulators as capacitors of interspecies variations in gene expression. Mol Syst Biol.

[16] Elowitz MB, Levine AJ, Siggia ED, Swain PS. Stochastic Gene Expression in a Single Cell. Science. 2002;297(5584):1183–6. 10.1126/science.1070919 12183631

[17] Kærn M, Elston TC, Blake WJ, Collins JJ. Stochasticity in gene expression: from theories to phenotypes. Nat Rev Genet. 2005;6(6):451–64. 10.1038/nrg1615 15883588

[18] Raj A, van Oudenaarden A. Nature, nurture, or chance: stochastic gene expression and its consequences. Cell. 2008;135(2):216–26. 10.1016/j.cell.2008.09.050 PMC3118044 18957198

[19] Cortijo S, Aydin Z, Ahnert S, Locke JC. Widespread inter-individual gene expression variability in *Arabidopsis thaliana*. Mol Syst Biol. 2019;15(1):e8591. 10.15252/msb.20188591 PMC6346214 30679203

[20] Eling N, Morgan MD, Marioni JC (2019). Challenges in measuring and understanding biological noise. Nat Rev Genet.

[21] Becskei A, Kaufmann BB, van Oudenaarden A (2005). Contributions of low molecule number and chromosomal positioning to stochastic gene expression. Nat Genet.

[22] Waddington CH (1942). Canalization of development and genetic assimilation of acquired characters. Nature..

[23] Lehner B (2010). Genes confer similar robustness to environmental, stochastic, and genetic perturbations in yeast. PLoS One.

[24] Meiklejohn CD, Hartl DL (2002). A single mode of canalization. Trends Ecol Evol.

[25] Alpern D, Gardeux V, Russeil J, Mangeat B, Meireles-Filho ACA, Breysse R (2019). BRB-seq: ultra-affordable high-throughput transcriptomics enabled by bulk RNA barcoding and sequencing. Genome Biol.

[26] Schep AN, Adryan B (2013). A comparative analysis of transcription factor expression during metazoan embryonic development. PLoS One.

[27] Lehner B (2008). Selection to minimise noise in living systems and its implications for the evolution of gene expression. Mol Syst Biol.

[28] Newman JRS, Ghaemmaghami S, Ihmels J, Breslow DK, Noble M, DeRisi JL (2006). Single-cell proteomic analysis of S. cerevisiae reveals the architecture of biological noise. Nature..

[29] Tadros W, Lipshitz HD. The maternal-to-zygotic transition: a play in two acts. Development. 2009;136(18):3033–42. 10.1242/dev.033183 19700615

[30] Levin M, Anavy L, Cole AG, Winter E, Mostov N, Khair S (2016). The mid-developmental transition and the evolution of animal body plans. Nature..

[31] Dreos R, Ambrosini G, Périer RC, Bucher P (2015). The Eukaryotic Promoter Database: expansion of EPDnew and new promoter analysis tools. Nucleic Acids Res.

[32] Siepel A, Bejerano G, Pedersen JS, Hinrichs AS, Hou M, Rosenbloom K (2005). Evolutionarily conserved elements in vertebrate, insect, worm, and yeast genomes. Genome Res.

[33] Benayoun BA, Pollina EA, Ucar D, Mahmoudi S, Karra K, Wong ED (2014). H3K4me3 breadth is linked to cell identity and transcriptional consistency. Cell..

[34] Wu S, Li K, Li Y, Zhao T, Li T, Yang Y-F (2017). Independent regulation of gene expression level and noise by histone modifications. PLoS Comput Biol.

[35] Nicolas D, Zoller B, Suter DM, Naef F (2018). Modulation of transcriptional burst frequency by histone acetylation. Proc Natl Acad Sci U S A.

[36] Weinberger L, Voichek Y, Tirosh I, Hornung G, Amit I, Barkai N (2012). Expression noise and acetylation profiles distinguish HDAC functions. Mol Cell.

[37] Faure AJ, Schmiedel JM, Lehner B (2017). Systematic analysis of the determinants of gene expression noise in embryonic stem cells. Cell Syst.

[38] Xiao L, Zhao Z, He F, Du Z (2019). Multivariable regulation of gene expression plasticity in metazoans. Open Biol.

[39] Nègre N, Brown CD, Ma L, Bristow CA, Miller SW, Wagner U, et al. A cis-regulatory map of the Drosophila genome. Nature. 2011;471(7339):527–31. 10.1038/nature09990 PMC3179250 21430782

[40] Nozaki T, Yachie N, Ogawa R, Kratz A, Saito R, Tomita M. Tight associations between transcription promoter type and epigenetic variation in histone positioning and modification. BMC Genomics. 2011;12(1):416. 10.1186/1471-2164-12-416 PMC3170308 21846408

[41] Sigalova OM, Shaeiri A, Forneris M, Furlong EE, Zaugg JB. Predictive features of gene expression variation reveal mechanistic link with differential expression. Mol Syst Biol. 2020;16(8):e9539. 10.15252/msb.20209539 PMC7411568 32767663

[42] Karlić R, Chung H-R, Lasserre J, Vlahovicek K, Vingron M (2010). Histone modification levels are predictive for gene expression. Proc Natl Acad Sci U S A.

[43] Gout J-F, Kahn D, Duret L, Paramecium Post-Genomics Consortium PP-G (2010). The relationship among gene expression, the evolution of gene dosage, and the rate of protein evolution. PLoS Genet.

[44] Coronado-Zamora M, Salvador-Martínez I, Castellano D, Barbadilla A, Salazar-Ciudad I (2019). Adaptation and conservation throughout the Drosophila melanogaster life-cycle. Genome Biol Evol.

[45] Piasecka B, Lichocki P, Moretti S, Bergmann S, Robinson-Rechavi M (2013). The hourglass and the early conservation models—co-existing patterns of developmental constraints in vertebrates. PLoS Genet.

[46] Wagner GP, Booth G, Bagheri-Chaichian H. A Population Genetic Theory of Canalization. Evolution. 1997;51(2):329-47. 10.1111/j.1558-5646.1997.tb02420.x 28565347

[47] Dobin A, Davis CA, Schlesinger F, Drenkow J, Zaleski C, Jha S (2013). STAR: ultrafast universal RNA-seq aligner. Bioinformatics..

[48] Zerbino DR, Achuthan P, Akanni W, Amode MR, Barrell D, Bhai J (2018). Ensembl 2018. Nucleic Acids Res.

[49] Anders S, Pyl PT, Huber W (2015). HTSeq--a Python framework to work with high-throughput sequencing data. Bioinformatics..

[50] Lun ATL, McCarthy DJ, Marioni JC (2016). A step-by-step workflow for low-level analysis of single-cell RNA-seq data. F1000Res.

[51] Robinson MD, McCarthy DJ, Smyth GK (2010). edgeR: a Bioconductor package for differential expression analysis of digital gene expression data. Bioinformatics..

[52] Law CW, Chen Y, Shi W, Smyth GK (2014). voom: precision weights unlock linear model analysis tools for RNA-seq read counts. Genome Biol.

[53] Johnson WE, Li C, Rabinovic A (2007). Adjusting batch effects in microarray expression data using empirical Bayes methods. Biostatistics..

[54] Kolodziejczyk AA, Kim JK, Tsang JCH, Ilicic T, Henriksson J, Natarajan KN (2015). Single cell RNA-sequencing of pluripotent states unlocks modular transcriptional variation. Cell Stem Cell.

[55] Avilés-Pagán EE, Orr-Weaver TL (2018). Activating embryonic development in Drosophila. Semin Cell Dev Biol.

[56] Raser JM, O’Shea EK (2005). Noise in gene expression: origins, consequences, and control. Science..

[57] Tung P-Y, Blischak JD, Hsiao CJ, Knowles DA, Burnett JE, Pritchard JK (2017). Batch effects and the effective design of single-cell gene expression studies. Sci Rep.

[58] Barroso GV, Puzovic N, Dutheil JY (2018). The evolution of gene-specific transcriptional noise is driven by selection at the pathway level. Genetics..

[59] Quinlan AR, Hall IM (2010). BEDTools: a flexible suite of utilities for comparing genomic features. Bioinformatics..

[60] Alexa A, Rahnenfuhrer J, Lengauer T (2006). Improved scoring of functional groups from gene expression data by decorrelating GO graph structure. Bioinformatics..

[61] Kim S (2015). ppcor: an R package for a fast calculation to semi-partial correlation coefficients. Commun Stat Appl methods.

[62] Liu J, Robinson-Rechavi M (2018). Developmental constraints on genome evolution in four bilaterian model species. Genome Biol Evol..

[63] Domazet-Loso T, Tautz D (2010). A phylogenetically based transcriptome age index mirrors ontogenetic divergence patterns. Nature..

[64] Carbon S, Ireland A, Mungall CJ, Shu S, Marshall B, Lewis S (2009). AmiGO: online access to ontology and annotation data. Bioinformatics..

[65] Paris M, Villalta JE, Eisen MB, Lott SE. Sex bias and maternal contribution to gene expression divergence in Drosophila blastoderm embryos. PLoS Genet. 2015;11(10):e1005592. 10.1371/journal.pgen.1005592 PMC4618353 26485701

[66] Hoskins RA, Landolin JM, Brown JB, Sandler JE, Takahashi H, Lassmann T (2011). Genome-wide analysis of promoter architecture in Drosophila melanogaster. Genome Res.

[67] Chen W-H, Lu G, Chen X, Zhao X-M, Bork P (2017). OGEE v2: an update of the online gene essentiality database with special focus on differentially essential genes in human cancer cell lines. Nucleic Acids Res.

[68] Liu J, Frochaux M, Gardeux V, Deplancke B, Robinson-Rechavi M. Inter-embryo gene expression variability recapitulates the hourglass pattern of evo-devo. NCBI accession number GSE128370. 2020. https://www.ncbi.nlm.nih.gov/geo/query/acc.cgi?acc=GSE128370. 10.1186/s12915-020-00842-z PMC7502200 32950053

