# Supplementary material for: Inter-embryo gene expression variability recapitulates the hourglass pattern of evo-devo
Source: BMC Biol. 2020 Sep 19;18:129. doi: 10.1186/s12915-020-00842-z (PMC7502200; doi:10.1186/s12915-020-00842-z)
Supplement: Supplementary file 1 — Additional file 1: Figure S1-S18. Figure S1. Relationship between uniquely mapped reads and expressed genes. Figure S2. Proportion of retained samples in each development stage. Figure S3. Evidence that the samples from the small cluster are unfertilized eggs. Figure S4. Relationship between average expression and coefficient of variation at each stage. Figure S5. Relationship between average expression and distance to median at each stage. Figure S6. Relationship between average expression and adjusted SD at each stage. Figure S7. Relationship between expression variability and protein importance. Figure S8. Variation of expression variability across development using alternate measures of variability. Figure S9. Bootstrap analysis of the variability calculation. Figure S10. Random sampling analysis of expression variability. Figure S11. Expression variability pattern for genes without maternally expressed genes. Figure S12. Expression variability pattern for genes expressed at all stages. Figure S13. Promoter sequence conservation across embryogenesis for different definitions of promoter width. Figure S14. Relationship between promoter shape index and histone modification signal. Figure S15. Relationship between promoter shape index and expression variability. Figure S16. Multidimensional scaling analysis for all samples. Figure S17. Mapping of X/autosome gene expression ratios to the multidimensional scaling analysis plot. Figure S18. Detection of stage specific genes. [file 12915_2020_842_MOESM1_ESM.pdf]

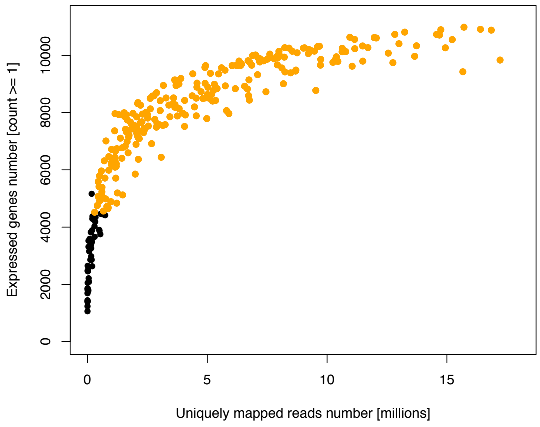

Figure S1: Relationship between uniquely mapped reads and expressed genes

Each dot represents one sample. The black dots indicate low quality samples with <4500 expressed genes or with <0.3 million uniquely mapped reads. The 239 orange colored samples were retained for downstream analysis ("high quality samples").

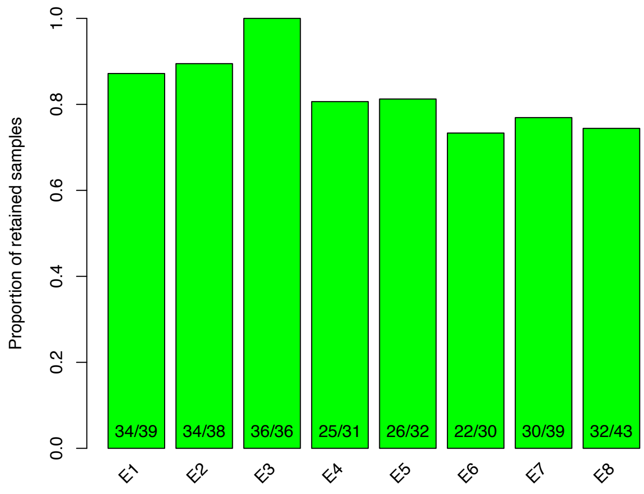

Figure S2: Proportion of retained samples in each development stage

The number of retained samples and of total samples in each stage is indicated in the bottom of each bar.

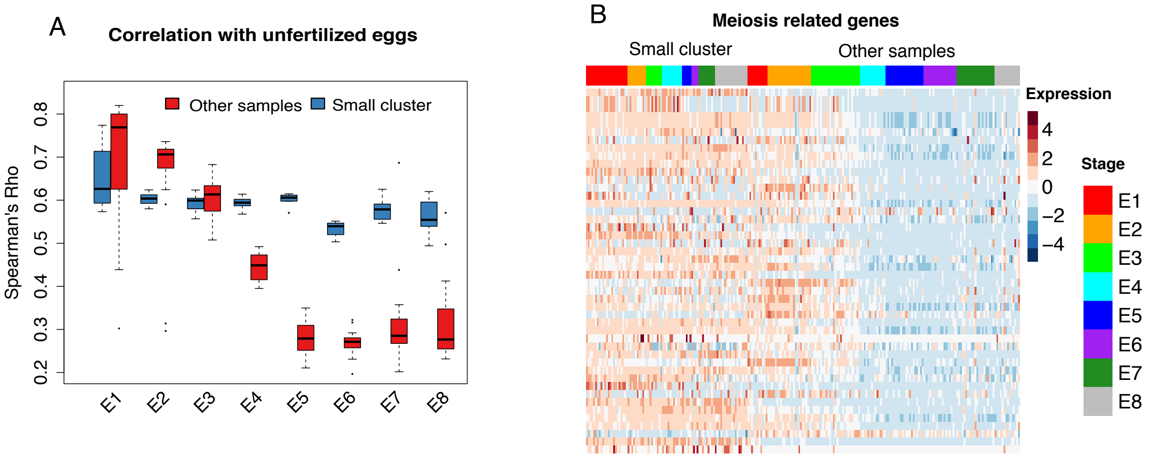

Figure S3: Evidence that the samples from the small cluster are unfertilized eggs

A. Boxplot of Spearman's correlation coefficients (rho) of expression between individual unfertilized eggs and each sample from the small cluster or from the large cluster, showing that the small cluster has an expression profile of unfertilized eggs. The lower and upper intervals indicated by the dashed lines ("whiskers") represent 1.5 times the interquartile range (IQR), and the box shows the lower and upper intervals of IQR together with the median.

B. Expression heat map of meiosis related genes across all samples, showing that their expression decreases over development for the large cluster, but is high in all samples of the small cluster, consistent with unfertilized eggs.

For testing of an alternative explanation of the two clusters as being males and females, see Figure S14.

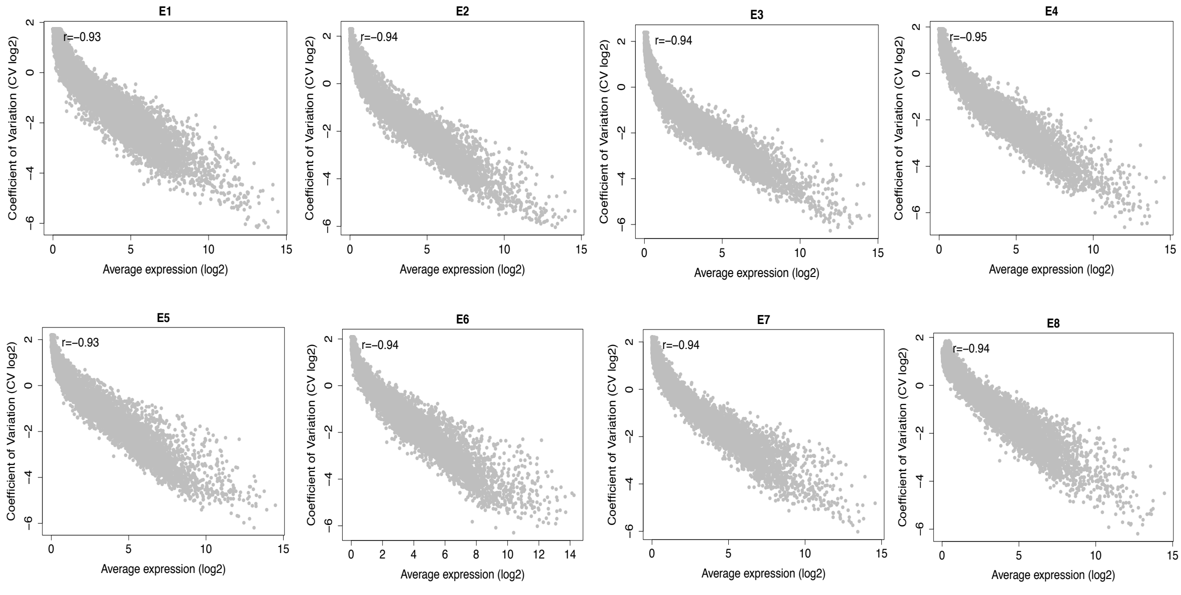

Figure S4: Relationship between average expression and coefficient of variation at each stage

Pearson’s correlation between average expression and coefficient of variation in each development stage is indicated in the top left of each subfigure.

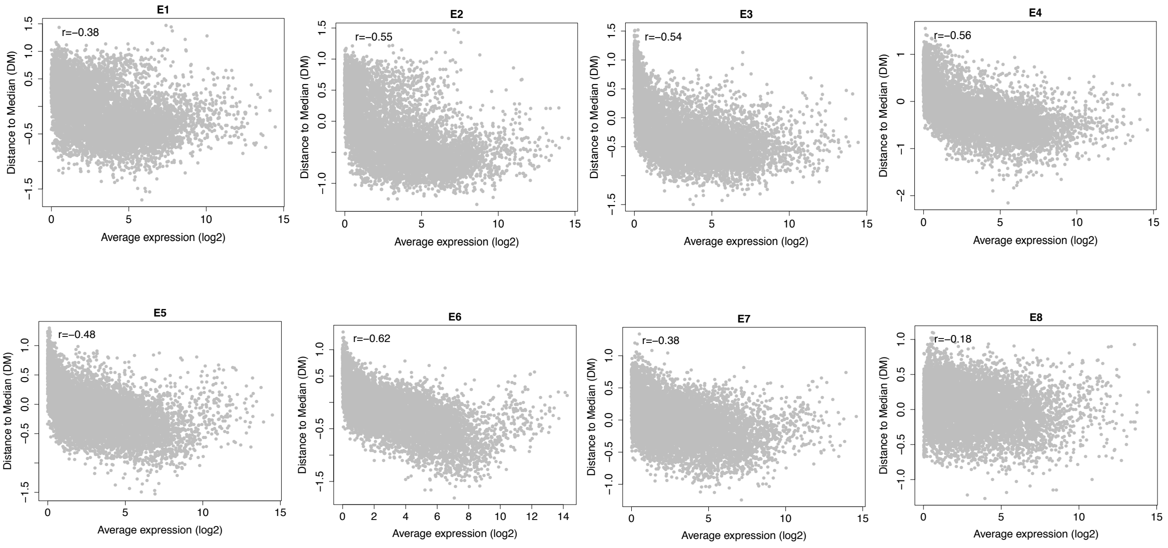

Figure S5: Relationship between average expression and distance to median at each stage

Pearson’s correlation between average expression and distance to median in each development stage is indicated in the top left of each subfigure.

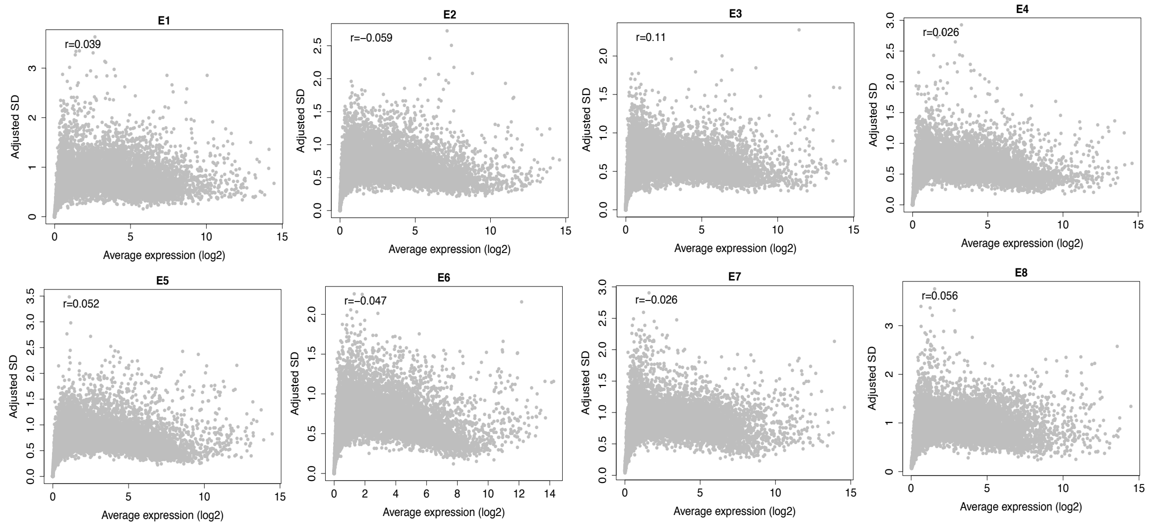

Figure S6: Relationship between average expression and adjusted SD at each stage

Pearson’s correlation between average expression and adjusted SD in each development stage is indicated in the top left of each subfigure.

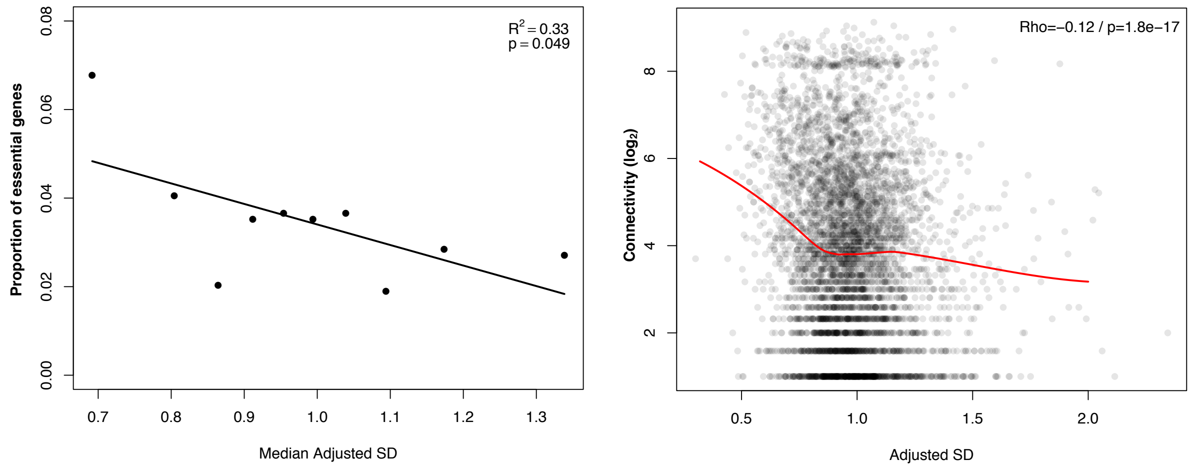

Figure S7: Relationship between expression variability and protein importance

We used the average variability across all development stages.

A. We split genes into 10 equally sized bins based on expression variability. The proportion of essential genes was fit by regression (the first degree of polynomial), whose R^2 and *p*-value are indicated in the top-left corner of each graph. The median expression variability of each bin was plotted on the x-axis.

B. Spearman's correlation between connectivity in a protein-protein interaction network and expression variability. The coefficient and *p*-value are indicated in the top-right. Loess regression lines are plotted in red.

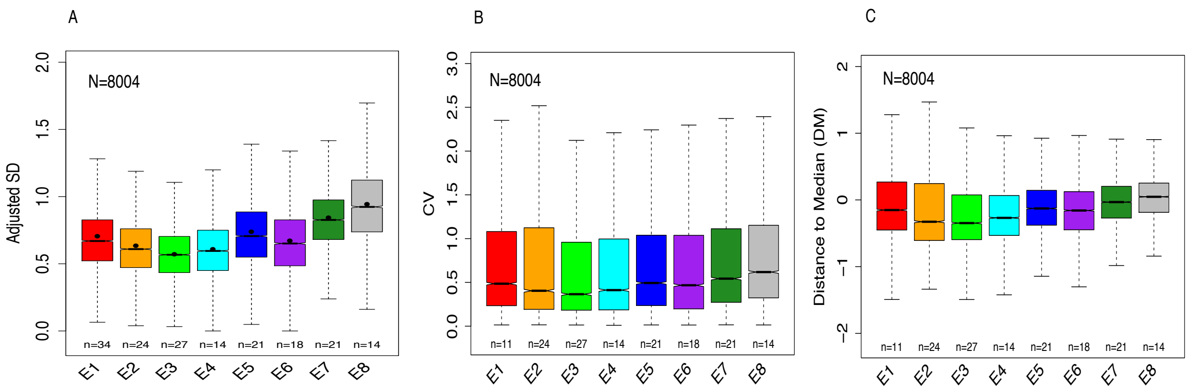

Figure S8: Variation of expression variability across development using alternate measures of variability

A. Variability measured by adjusted SD; unlike in Figure 2, the variability in E1 was calculated using all samples from both small and large clusters.

B. Variability measured by coefficient of variation (CV).

C. Variability measured by distance to median (DM).

The legend is the same as for Figure 2. We performed pairwise Wilcoxon test between any two stages to test the significance. The multiple test corrected *p*-values (Benjamini–Hochberg method) are shown in Additional file 2: Tables S6, S7 and S8.

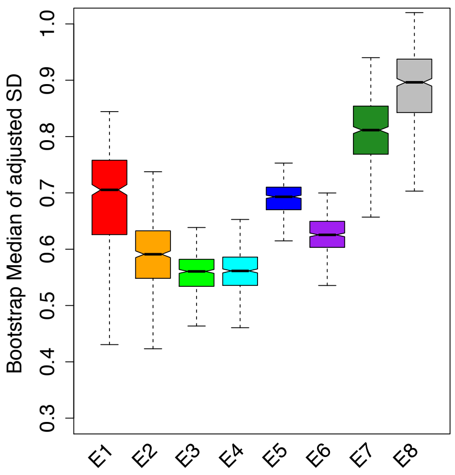

Figure S9: Bootstrap analysis of the variability calculation

We performed pairwise Wilcoxon test between any two stages to test the significance. The multiple test corrected *p*-values (Benjamini–Hochberg method) are shown in Additional file 2: Table S9.

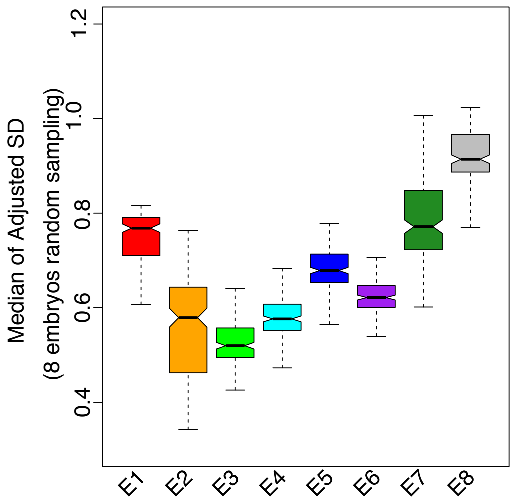

Figure S10: Random sampling analysis of expression variability

Distribution of the median adjusted SD over all genes, for 500 random resampling of 8 samples per time point. We performed pairwise Wilcoxon test between any two stages to test the significance. The multiple test corrected p-values (Benjamini–Hochberg method) are shown in Additional file 2: Table S10.

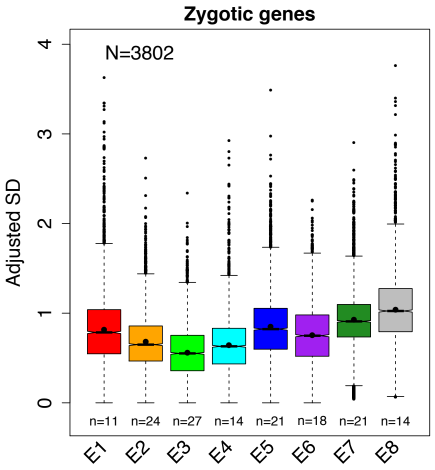

Figure S11: Expression variability pattern for genes without maternally expressed genes

The number of individual samples used in each development stage is indicated below each box. The number of genes analyzed is indicated in the top-left corner of each plot. The black dot in each box indicates the mean. The multiple test corrected p-values (Benjamini–Hochberg method) are shown in Additional file 2: Table S11.

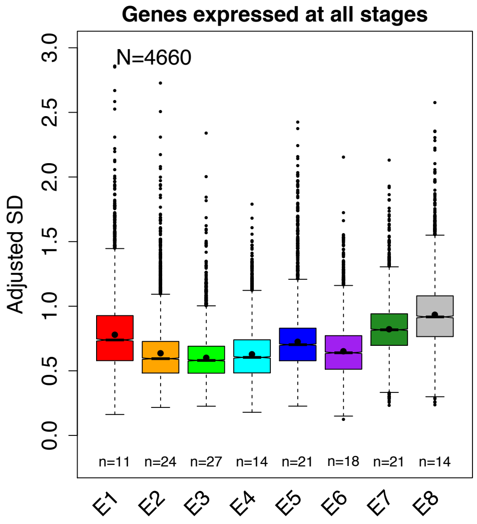

Figure S12: Expression variability pattern for genes expressed at all stages

The number of individual samples used in each development stage is indicated below each box. The number of genes analyzed is indicated in the top-left corner of each plot. The black dot in each box indicates the mean. The multiple test corrected p-values (Benjamini–Hochberg method) are shown in Additional file 2: Table S12.

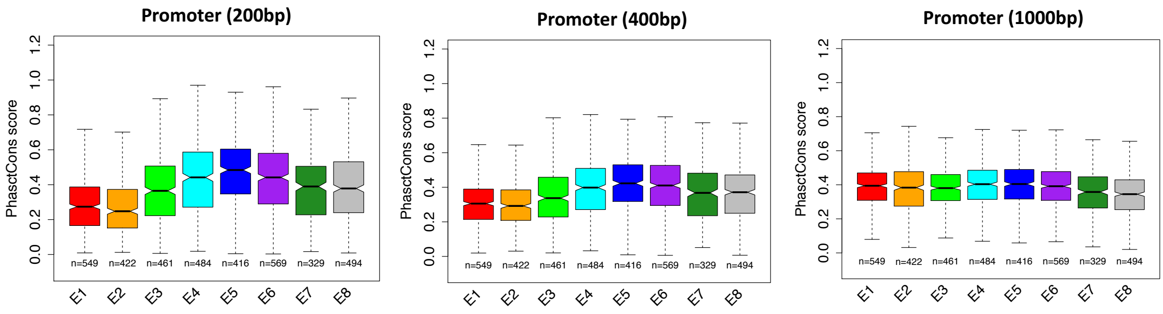

Figure S13: Promoter sequence conservation across embryogenesis for different definitions of promoter width

The figure legend is the same as in Figure 3A.

200bp promoter: 100 bp upstream transcription TSS to 100 bp downstream of the TSS.

400 bp promoter: 200 bp upstream transcription TSS to 200 bp downstream of the TSS.

1000 bp promoter: 500 bp upstream transcription TSS to 500 bp downstream of the TSS.

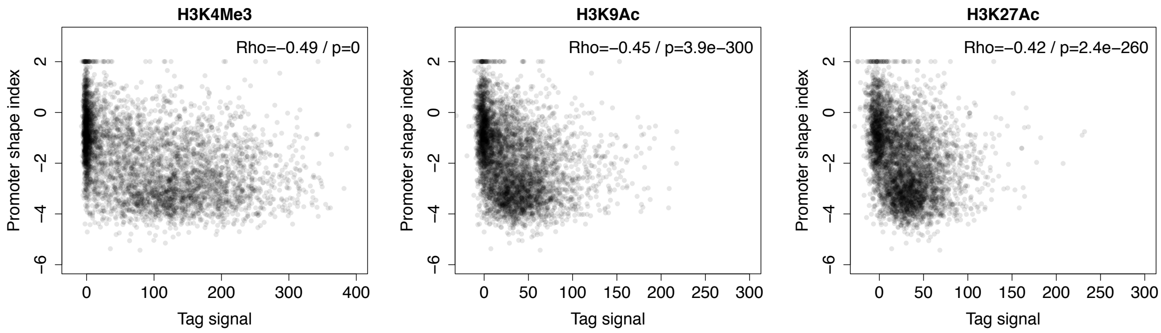

Figure S14: Relationship between promoter shape index and histone modification signal

Spearman's correlation between promoter shape index and histone modification signal. We used the average signal across all development stages. The coefficient and *p*-value are indicated in the top-right.

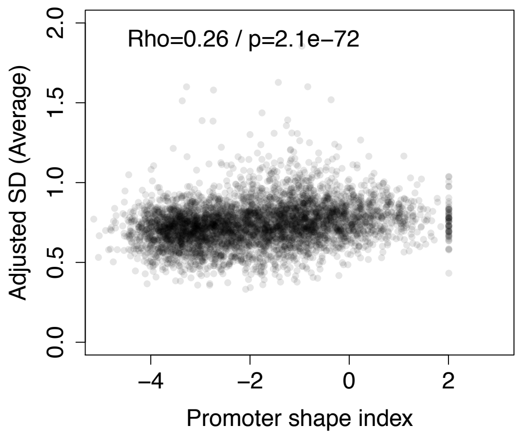

Figure S15: Relationship between promoter shape index and expression variability

Spearman's correlation between promoter shape index and expression variability. Lower promoter shape index means broader promoter. We used the average variability across all development stages. The coefficient and *p*-value are indicated in the top-left.

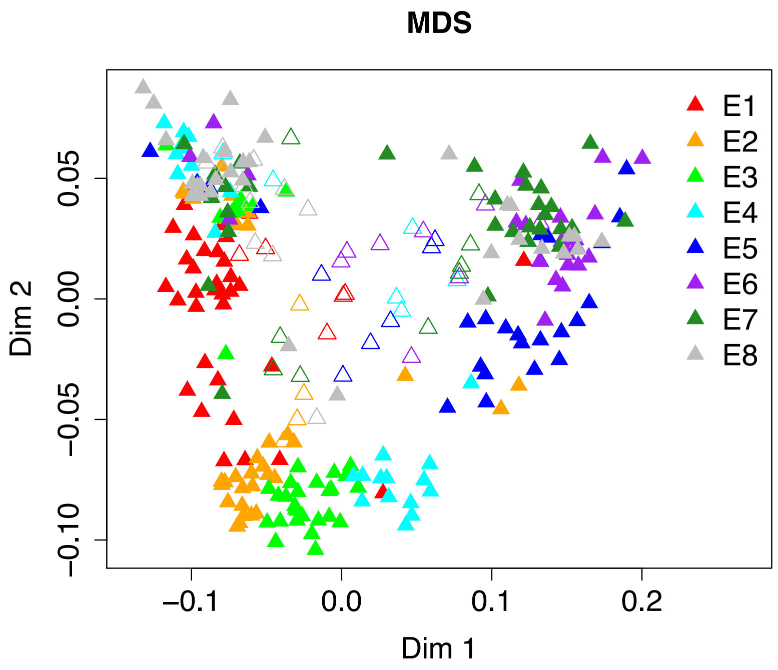

Figure S16: Multidimensional scaling analysis for all samples

Different colors indicate different stages. The solid triangles represent high quality samples according to Figure S1; the hollow triangles represent low quality samples which were discarded.

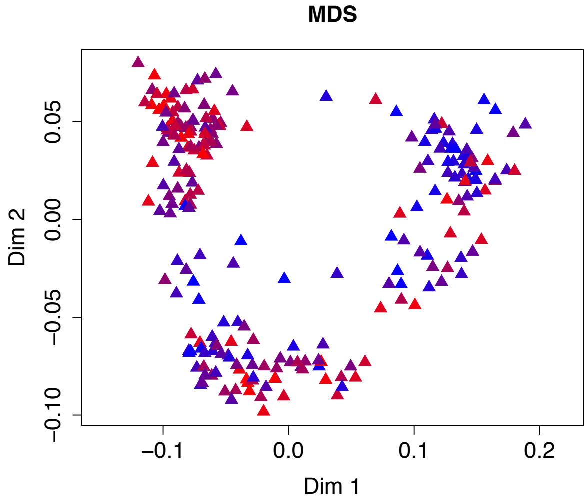

Figure S17: Mapping of X/autosome gene expression ratios to the multidimensional scaling analysis plot

We calculated the ratio of mean expression between genes from the X chromosome and from the autosomes for each sample. Red represents high ratio, blue represents low ratio. For Drosophila, dosage compensation is achieved by increasing expression of X chromosome genes in males. Since the dosage compensation is still incomplete during development, females should have a higher ratio of mean expression between genes from the X chromosome and from the autosomes. Here, we found both high ratio samples and low ratio samples are well mixed in both the cluster and large clusters. Thus, we reject the hypothesis that the two different clusters are due to sex.

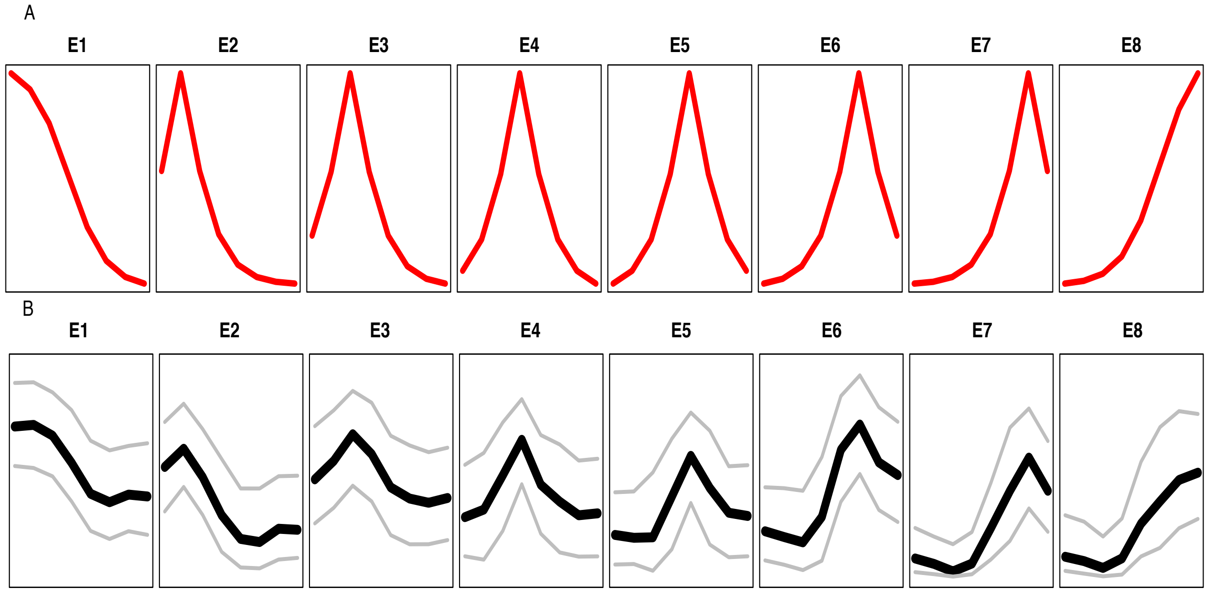

Figure S18: Detection of stage specific genes

A. The artificial expression profile.

The expression of identified stage specific genes. The bold black line represents the median expression, the two gray lines represent 25th and 75th quantiles of expression, respectively.
