# Supplementary material for: Inter-embryo gene expression variability recapitulates the hourglass pattern of evo-devo
Source: BMC Biol. 2020 Sep 19;18:129. doi: 10.1186/s12915-020-00842-z (PMC7502200; doi:10.1186/s12915-020-00842-z)
Supplement: Supplementary file 2 — Additional file 2: Tables. S1-S13. Table S1-S12. multiple test corrected p-values of variability (adjusted SD) comparison between any two stages. Table S13. RNA-seq library information. [file 12915_2020_842_MOESM2_ESM.pdf]

Table S1: variability (adjusted SD) comparison between any two stages

Wilcoxon test, multiple test corrected *p*-values (Benjamini–Hochberg method).

Stages	E1	E2	E3	E4	E5	E6	E7
E2	3.11e-180						
E3	0.00e+00	6.17e-43					
E4	1.21e-247	5.24e-08	2.49e-16				
E5	1.06e-13	2.57e-118	4.14e-292	1.99e-175			
E6	1.93e-92	3.99e-17	2.62e-105	8.75e-43	1.14e-44		
E7	2.57e-78	0.00e+00	0.00e+00	0.00e+00	3.06e-181	0.00e+00	
E8	6.64e-268	0.00e+00	0.00e+00	0.00e+00	0.00e+00	0.00e+00	3.14e-106

Table S2: promoter sequence conservation comparison between any two stages

Wilcoxon test, multiple test corrected *p*-values (Benjamini–Hochberg method).

Stages	E1	E2	E3	E4	E5	E6	E7
E2	5.02e-01						
E3	1.70e-11	4.66e-12					
E4	5.57e-29	1.96e-28	4.06e-06				
E5	1.07e-35	7.75e-35	2.93e-10	8.85e-02			
E6	2.18e-37	3.23e-36	5.29e-09	4.19e-01	3.13e-01		
E7	1.89e-11	5.11e-12	7.91e-01	2.09e-05	2.13e-09	8.87e-08	
E8	2.28e-21	1.72e-21	2.18e-02	9.67e-03	7.81e-06	1.79e-04	6.34e-02

Table S3: proximal promoter H3K4Me3 signal (Z score) comparison between any two stages

Wilcoxon test, multiple test corrected *p*-values (Benjamini–Hochberg method).

Satges	0-4h	4-8h	8-12h	12-16h	16-20h
4-8h	2.39e-36				
8-12h	3.71e-93	5.51e-13			
12-16h	6.35e-35	6.84e-01	1.28e-17		
16-20h	2.29e-09	6.05e-08	4.17e-47	7.73e-11	
20-24h	3.10e-88	2.08e-118	8.88e-197	1.40e-161	9.07e-182

Table S4: proximal promoter H3K9Ac signal (Z score) comparison between any two stages

Wilcoxon test, multiple test corrected *p*-values (Benjamini–Hochberg method).

Satges	0-4h	4-8h	8-12h	12-16h	16-20h
4-8h	1.73e-06				
8-12h	2.17e-40	3.75e-16			
12-16h	9.49e-01	1.71e-07	2.17e-40		
16-20h	4.50e-78	2.03e-97	1.23e-171	1.41e-67	
20-24h	7.31e-145	3.75e-148	1.11e-230	8.16e-121	1.13e-28

Table S5: proximal promoter H3K27Ac signal (Z score) comparison between any two stages

Wilcoxon test, multiple test corrected *p*-values (Benjamini–Hochberg method).

Satges	0-4h	4-8h	8-12h	12-16h	16-20h
4-8h	2.45e-03				
8-12h	7.91e-141	2.12e-88			
12-16h	6.90e-01	1.85e-04	4.74e-127		
16-20h	8.98e-01	3.97e-03	6.20e-147	6.87e-01	
20-24h	2.97e-103	8.33e-85	6.08e-292	1.31e-57	1.89e-165

Table S6: variability (adjusted SD) comparison between any two stages

Here, the E1 stage also contains samples from the small clusters. Wilcoxon test, multiple test corrected *p*-values (Benjamini–Hochberg method).

Stages	E1	E2	E3	E4	E5	E6	E7
E2	5.49e-58						
E3	6.24e-202	2.55e-46					
E4	1.02e-96	1.57e-07	1.60e-18				
E5	9.25e-19	1.32e-124	3.41e-303	6.09e-175			
E6	2.56e-09	3.21e-19	6.02e-112	2.66e-43	1.86e-43		
E7	0.00e+00	0.00e+00	0.00e+00	0.00e+00	1.56e-163	0.00e+00	
E8	0.00e+00	0.00e+00	0.00e+00	0.00e+00	0.00e+00	0.00e+00	6.72e-104

Table S7: variability (coefficient of variation) comparison between any two stages

Wilcoxon test, multiple test corrected *p*-values (Benjamini–Hochberg method).

Stages	E1	E2	E3	E4	E5	E6	E7
E2	9.30e-9						
E3	1.35e-25	5.29e-05					
E4	1.24e-15	3.32e-02	4.33e-02				
E5	6.79e-01	3.64e-08	1.22e-24	9.45e-15			
E6	1.70e-05	1.94e-01	1.69e-08	3.31e-04	9.93e-05		
E7	1.87e-08	9.49e-30	3.66e-60	9.84e-43	1.76e-09	2.14e-22	
E8	3.55e-28	3.96e-59	3.97e-103	1.39e-78	1.11e-31	1.91e-50	1.40e-08

Table S8: variability (distance to median) comparison between any two stages

Wilcoxon test, multiple test corrected *p*-values (Benjamini–Hochberg method).

Stages	E1	E2	E3	E4	E5	E6	E7
E2	5.64e-51						
E3	4.83e-94	3.00e-04					
E4	3.57e-54	1.13e-01	3.90e-10				
E5	9.54e-01	2.03e-61	1.09e-119	4.91e-70			
E6	1.58e-11	1.82e-18	9.34e-52	3.13e-24	2.71e-11		
E7	2.41e-30	1.35e-154	5.48e-265	5.28e-203	3.12e-44	5.15e-87	
E8	9.09e-98	1.10e-251	0.00e+00	0.00e+00	9.77e-148	2.36e-210	1.22e-37

Table S9: bootstrapped median variability comparison between any two stages

Wilcoxon test, multiple test corrected *p*-values (Benjamini–Hochberg method).

Stages	E1	E2	E3	E4	E5	E6	E7
E2	1.60e-66						
E3	7.73e-99	3.94e-22					
E4	2.26e-96	1.38e-18	2.41e-18				
E5	1.14e-02	4.67e-134	1.20e-303	4.16e-163			
E6	1.50e-40	1.33e-27	2.62e-112	1.17e-111	3.11e-123		
E7	4.58e-90	2.51e-163	4.15e+00	4.15e-164	2.96e-141	1.05e-163	
E8	4.15e-139	1.05e-163	4.15e+00	4.15e-164	7.64e-159	1.20e-163	2.60e-59

Table S10: Random sampling variability comparison between any two stages

Wilcoxon test, multiple test corrected *p*-values (Benjamini–Hochberg method).

Stages	E1	E2	E3	E4	E5	E6	E7
E2	2.58e−48						
E3	7.50e−60	2.06e−03					
E4	7.11e−53	4.66e−01	2.17e−23				
E5	5.75e−22	3.13e−35	1.17e−62	9.66e−57			
E6	5.59e−42	4.44e−09	7.67e−50	7.27e−27	2.89e−32		
E7	6.10e−04	4.12e−58	9.24e−66	7.37e−65	1.47e−34	8.27e−59	
E8	7.37e−55	4.30e−66	4.30e−66	4.30e−66	2.47e−65	4.30e−66	6.38e−38

Table S11: variability (adjusted SD) comparison between any two stages (for genes without maternally expressed genes)

Wilcoxon test, multiple test corrected *p*-values (Benjamini–Hochberg method).

Stages	E1	E2	E3	E4	E5	E6	E7
E2	3.25e−51						
E3	4.81e−175	2.21e−53					
E4	2.46e−78	3.42e−05	2.09e−27				
E5	1.14e−05	4.81e−92	1.01e−247	3.48e−127			
E6	8.65e−07	1.11e−26	5.08e−135	5.46e−48	3.46e−22		
E7	2.31e−51	2.49e−227	0.00e+00	5.31e−280	3.03e−28	4.10e−98	
E8	1.78e−140	0.00e+00	0.00e+00	0.00e+00	2.35e−109	6.47e−212	7.64e−45

Table S12: variability (adjusted SD) comparison between any two stages (for genes expressed at all stages)

Wilcoxon test, multiple test corrected *p*-values (Benjamini–Hochberg method).

Stages	E1	E2	E3	E4	E5	E6	E7
E2	3.57e-175						
E3	3.05e-254	2.92e-06					
E4	6.57e-170	2.39e-01	7.21e-10				
E5	2.05e-17	3.71e-125	1.92e-200	7.95e-113			
E6	9.89e-105	1.45e-17	2.93e-45	2.38e-13	2.87e-52		
E7	9.01e-47	0.00e+00	0.00e+00	0.00e+00	1.97e-163	0.00e+00	
E8	3.11e-199	0.00e+00	0.00e+00	0.00e+00	0.00e+00	0.00e+00	2.19e-107

Table S13: RNA-seq library information

	Library E1_1	
stage	position in 96-well	sample barcode
embryo2-3h	A2	AAATCA
embryo2-3h	B12	GTTAAA
embryo2-3h	C6	TATAAG
embryo2-3h	D12	GAAGAC
embryo2-3h	D2	TTACTA
embryo2-3h	D4	TTCATA
embryo5-6h	B10	GAATAT
embryo5-6h	B7	CATAAA
embryo5-6h	C10	TCTAAT
embryo5-6h	D10	CATCAG
embryo5-6h	D9	ATCGAG
embryo8-9h	A12	ATAGAT
embryo8-9h	A3	AACATA
embryo8-9h	B8	CTAAAA
embryo8-9h	B9	CTTAAT
embryo8-9h	C11	TGAATA
embryo8-9h	C3	TAATAG
embryo8-9h	C4	TACAAA
embryo8-9h	C5	TAGAAT
embryo8-9h	C8	TATTAC
embryo8-9h	D5	TTGTTA
embryo8-9h	D7	TTTTAG
embryo11-12h	A1	AAACAT
embryo11-12h	A11	ATAAGT
embryo11-12h	A4	AAGTTA
embryo11-12h	A5	AATATG
embryo11-12h	A6	AATGTA
embryo11-12h	A7	AATTTC
embryo11-12h	B5	ATTGTT
embryo11-12h	C7	TATCAT
embryo11-12h	C9	TCAAAA
embryo11-12h	D1	TTAACT
embryo11-12h	D6	TTTATG
embryo14-15h	A8	ACTAAA
embryo14-15h	A9	AGAATT
embryo14-15h	B1	ATATGA
embryo14-15h	B11	GATTAA
embryo14-15h	B4	ATTATC
embryo14-15h	C12	TGTATT
embryo14-15h	D11	CGACTT

embryo14-15h	D8	TTGGAT
embryo17-18h	B2	ATCTAT
embryo17-18h	B6	ATTTTG
embryo17-18h	C1	TAAAAC
embryo17-18h	C2	TAACAA
embryo20-21h	A10	AGTATA
embryo20-21h	D3	TTATCA
embryo23-24h	B3	ATGTAA

	Library E1_2	
stage	position in 96-well	sample barcode
embryo2-3h	E10	CACCGG
embryo2-3h	F2	CCCGAG
embryo2-3h	F3	CCGCAG
embryo2-3h	F7	CGCAGG
embryo2-3h	G10	GCGCCA
embryo2-3h	H11	TGCGCG
embryo2-3h	H5	GGGCGA
embryo2-3h	H8	GTCGCG
embryo5-6h	E2	GTCAAG
embryo5-6h	E6	ACCCGG
embryo5-6h	G11	GCGGCT
embryo5-6h	H7	GGTCCC
embryo8-9h	E1	GGACAT
embryo8-9h	E9	AGGCGG
embryo8-9h	F8	CGCCTC
embryo8-9h	G5	GAGGGC
embryo8-9h	G7	GCCCCT
embryo8-9h	H10	TCGCGG
embryo8-9h	H12	TGGGCC
embryo8-9h	H4	GGGACC
embryo8-9h	H9	GTGGGG
embryo11-12h	E5	CTCTCC
embryo11-12h	E8	AGCCGC
embryo11-12h	F6	CGACGG
embryo14-15h	E12	CCACGC
embryo14-15h	E4	TTGTCC
embryo14-15h	E7	ACGCGC
embryo14-15h	F10	CGGAGC
embryo14-15h	F9	CGCGTG
embryo14-15h	G1	CGTCGC
embryo14-15h	G8	GCCGCA
embryo17-18h	G4	GACGGG

embryo17-18h	G6	GCAGGC
embryo17-18h	G9	GCCTGC
embryo17-18h	H1	GCTGGG
embryo17-18h	H3	GGCGCT
embryo17-18h	H6	GGGGGT
embryo20-21h	E11	CAGCGC
embryo20-21h	F1	CCCCAC
embryo20-21h	F11	CGGCTG
embryo20-21h	F12	CGGGTC
embryo20-21h	F4	CCGGGT
embryo20-21h	F5	CCTCGG
embryo20-21h	G12	GCGTGG
embryo20-21h	H2	GGCACG
embryo23-24h	E3	TCCAAC
embryo23-24h	G2	CTCCGC
embryo23-24h	G3	CTGGGC

Library E2

stage	position in 96-well	sample barcode
embryo2-3h	B6	ATTTTG
embryo2-3h	C12	TGTATT
embryo2-3h	D10	CATCAG
embryo2-3h	D2	TTACTA
embryo2-3h	D6	TTTATG
embryo2-3h	E1	GGACAT
embryo2-3h	E10	CACCGG
embryo2-3h	E11	CAGCGC
embryo2-3h	H1	GCTGGG
embryo2-3h	H11	TGCGCG
embryo5-6h	A11	ATAAGT
embryo5-6h	A3	AACATA
embryo5-6h	A4	AAGTTA
embryo5-6h	A7	AATTTC
embryo5-6h	A9	AGAATT
embryo5-6h	B3	ATGTAA
embryo5-6h	B7	CATAAA
embryo5-6h	B8	CTAAAA
embryo5-6h	D7	TTTTAG
embryo5-6h	D9	ATCGAG
embryo5-6h	E12	CCACGC
embryo5-6h	F10	CGGAGC
embryo5-6h	G4	GACGGG
embryo5-6h	G9	GCCTGC

embryo5-6h	H7	GGTCCC
embryo5-6h	H8	GTCGCG
embryo8-9h	A10	AGTATA
embryo8-9h	A6	AATGTA
embryo8-9h	B2	ATCTAT
embryo8-9h	C10	TCTAAT
embryo8-9h	D4	TTCATA
embryo8-9h	E4	TTGTCC
embryo8-9h	E7	ACGCGC
embryo8-9h	F2	CCCGAG
embryo8-9h	G5	GAGGGC
embryo8-9h	H12	TGGGCC
embryo8-9h	H9	GTGGGG
embryo11-12h	A8	ACTAAA
embryo11-12h	B10	GAATAT
embryo11-12h	B4	ATTATC
embryo11-12h	B9	CTTAAT
embryo11-12h	F11	CGGCTG
embryo11-12h	F12	CGGGTC
embryo11-12h	G10	GCGCCA
embryo11-12h	H2	GGCACG
embryo14-15h	C3	TAATAG
embryo14-15h	C6	TATAAG
embryo14-15h	C7	TATCAT
embryo14-15h	D1	TTAACT
embryo14-15h	D8	TTGGAT
embryo14-15h	E9	AGGCGG
embryo14-15h	G6	GCAGGC
embryo14-15h	G8	GCCGCA
embryo17-18h	A1	AAACAT
embryo17-18h	A5	AATATG
embryo17-18h	B1	ATATGA
embryo17-18h	B12	GTTAAA
embryo17-18h	C1	TAAAAC
embryo17-18h	C8	TATTAC
embryo17-18h	D5	TTGTTA
embryo17-18h	E2	GTCAAG
embryo17-18h	E5	CTCTCC
embryo17-18h	F1	CCCCAC
embryo17-18h	F6	CGACGG
embryo17-18h	G2	CTCCGC
embryo20-21h	A2	AAATCA
embryo20-21h	C4	TACAAA

embryo20-21h	E6	ACCCGG
embryo20-21h	F3	CCGCAG
embryo20-21h	F5	CCTCGG
embryo20-21h	F8	CGCCTC
embryo20-21h	G1	CGTCGC
embryo20-21h	G11	GCGGCT
embryo20-21h	H10	TCGCGG
embryo23-24h	A12	ATAGAT
embryo23-24h	B11	GATTAA
embryo23-24h	B5	ATTGTT
embryo23-24h	C11	TGAATA
embryo23-24h	C2	TAACAA
embryo23-24h	C5	TAGAAT
embryo23-24h	C9	TCAAAA
embryo23-24h	D11	CGACTT
embryo23-24h	D12	GAAGAC
embryo23-24h	D3	TTATCA
embryo23-24h	E3	TCCAAC
embryo23-24h	E8	AGCCGC
embryo23-24h	F4	CCGGGT
embryo23-24h	F7	CGCAGG
embryo23-24h	F9	CGCGTG
embryo23-24h	G12	GCGTGG
embryo23-24h	G3	CTGGGC
embryo23-24h	G7	GCCCCT
embryo23-24h	H3	GGCGCT
embryo23-24h	H4	GGGACC
embryo23-24h	H5	GGGCGA
embryo23-24h	H6	GGGGGT

stage	Library E3 position in 96-well	sample barcode
embryo2-3h	A10	AGTATA
embryo2-3h	A12	ATAGAT
embryo2-3h	A7	AATTTC
embryo2-3h	A8	ACTAAA
embryo2-3h	B2	ATCTAT
embryo2-3h	C1	TAAAAC
embryo2-3h	C8	TATTAC
embryo2-3h	C9	TCAAAA
embryo2-3h	D3	TTATCA
embryo2-3h	F11	CGGCTG

embryo2-3h	F4	CCGGGT
embryo2-3h	F5	CCTCGG
embryo2-3h	G12	GCGTGG
embryo2-3h	G4	GACGGG
embryo2-3h	H9	GTGGGG
embryo5-6h	A2	AAATCA
embryo5-6h	B1	ATATGA
embryo5-6h	B9	CTTAAT
embryo5-6h	D1	TTAACT
embryo5-6h	D4	TTCATA
embryo5-6h	D5	TTGTTA
embryo5-6h	D6	TTTATG
embryo5-6h	E4	TTGTCC
embryo5-6h	F12	CGGGTC
embryo5-6h	G2	CTCCGC
embryo5-6h	G3	CTGGGC
embryo5-6h	G7	GCCCCT
embryo5-6h	H10	TCGCGG
embryo8-9h	B3	ATGTAA
embryo8-9h	D7	TTTTAG
embryo8-9h	E12	CCACGC
embryo8-9h	F8	CGCCTC
embryo8-9h	H1	GCTGGG
embryo11-12h	A1	AAACAT
embryo11-12h	A9	AGAATT
embryo11-12h	B11	GATTAA
embryo11-12h	B8	CTAAAA
embryo11-12h	C5	TAGAAT
embryo11-12h	F1	CCCCAC
embryo11-12h	F3	CCGCAG
embryo11-12h	F7	CGCAGG
embryo11-12h	H4	GGGACC
embryo14-15h	A5	AATATG
embryo14-15h	B4	ATTATC

embryo14-15h	C10	TCTAAT
embryo14-15h	C3	TAATAG
embryo14-15h	F10	CGGAGC
embryo14-15h	F2	CCCGAG
embryo14-15h	G10	GCGCCA
embryo14-15h	H2	GGCACG
embryo14-15h	H5	GGGCGA
embryo17-18h	D11	CGACTT
embryo17-18h	E11	CAGCGC
embryo17-18h	E3	TCCAAC
embryo17-18h	E9	AGGCGG
embryo17-18h	F9	CGCGTG
embryo17-18h	H11	TGCGCG
embryo17-18h	H12	TGGGCC
embryo17-18h	H3	GGCGCT
embryo20-21h	A11	ATAAGT
embryo20-21h	A3	AACATA
embryo20-21h	A4	AAGTTA
embryo20-21h	B10	GAATAT
embryo20-21h	B5	ATTGTT
embryo20-21h	B6	ATTTTG
embryo20-21h	B7	CATAAA
embryo20-21h	C2	TAACAA

embryo20-21h	C4	TACAAA
embryo20-21h	D2	TTACTA
embryo20-21h	D9	ATCGAG
embryo20-21h	E2	GTCAAG
embryo20-21h	E5	CTCTCC
embryo20-21h	E6	ACCCGG
embryo20-21h	E7	ACGCGC
embryo20-21h	E8	AGCCGC
embryo20-21h	G11	GCGGCT
embryo20-21h	G6	GCAGGC
embryo20-21h	G9	GCCTGC
embryo20-21h	H7	GGTCCC
embryo23-24h	A6	AATGTA
embryo23-24h	B12	GTTAAA
embryo23-24h	C11	TGAATA
embryo23-24h	C12	TGTATT
embryo23-24h	C6	TATAAG
embryo23-24h	C7	TATCAT
embryo23-24h	D10	CATCAG
embryo23-24h	D12	GAAGAC
embryo23-24h	D8	TTGGAT
embryo23-24h	E1	GGACAT
embryo23-24h	E10	CACCGG

embryo23-24h	F6	CGACGG
embryo23-24h	G1	CGTCGC
embryo23-24h	G5	GAGGGC
embryo23-24h	G8	GCCGCA
embryo23-24h	H6	GGGGGT
embryo23-24h	H8	GTCGCG
